# Foliar‐applied manganese and phosphorus in deficient barley: Linking absorption pathways and leaf nutrient status

**DOI:** 10.1111/ppl.13761

**Published:** 2022-08-22

**Authors:** Maja Arsic, Daniel P. Persson, Jan K. Schjoerring, Lisbeth G. Thygesen, Enzo Lombi, Casey L. Doolette, Søren Husted

**Affiliations:** ^1^ Department of Plant and Environmental Sciences University of Copenhagen Frederiksberg C Denmark; ^2^ University of South Australia Future Industries Institute Mawson Lakes South Australia Australia; ^3^ Department of Geosciences and Natural Resource Management University of Copenhagen Frederiksberg C Denmark; ^4^ Present address: CSIRO Agriculture and Food, Queensland Biosciences Precinct St. Lucia Queensland Australia

## Abstract

Foliar fertilization delivers essential nutrients directly to plant tissues, reducing excessive soil fertilizer applications that can lead to eutrophication following nutrient leaching. Foliar nutrient absorption is a dynamic process affected by leaf surface structure and composition, plant nutrient status, and ion physicochemical properties. We applied multiple methods to study the foliar absorption behaviors of manganese (Mn) and phosphorus (P) in nutrient‐deficient spring barley (*Hordeum vulgare*) at two growth stages. Nutrient‐specific chlorophyll *a* fluorescence assays were used to visualize leaf nutrient status, while laser ablation‐inductively coupled plasma‐mass spectrometry (LA‐ICP‐MS) was used to visualize foliar absorption pathways for P and Mn ions. Rapid Mn absorption was facilitated by a relatively thin cuticle with a low abundance of waxes and a higher stomatal density in Mn‐deficient plants. Following absorption, Mn accumulated in epidermal cells and in the photosynthetically active mesophyll, enabling a fast (6 h) restoration of Mn‐dependent photosynthetic processes. Conversely, P‐deficient plants developed thicker cuticles and epidermal cell walls, which reduced the penetration of P across the leaf surface. Foliar‐applied P accumulated in trichomes and fiber cells above leaf veins without reaching the mesophyll and, as a consequence, no restoration of P‐dependent photosynthetic processes was observed. This study reveals new links between leaf surface morphology, foliar‐applied ion absorption pathways, and the restoration of affected physiological processes in nutrient‐deficient leaves. Understanding that ions may have different absorption pathways across the leaf surface is critical for the future development of efficient fertilization strategies for crops in nutrient‐limited soils.

## 1 INTRODUCTION

Unsustainable fertilization practices threaten the resilience of environmental systems and future food security due to excessive nutrient applications to soils at sowing. Plant nutrient uptake is often inefficient due to biological and chemical soil processes; approximately only 15%–30% of applied phosphorus (P) fertilizers are absorbed by plants in the year of application (Syers et al., 2008). Furthermore, soil erosion and nutrient runoff into water bodies cause eutrophication (Kopittke et al., 2019; Withers et al., 2014). Consequently, altered flows of nitrogen and P pose significant risks to the stability of key earth system functions (Campbell et al., 2017; Steffen et al., 2015). Foliar fertilization has the potential to become a more sustainable and cost‐effective supplementary strategy to classic soil fertilization. This is already a common approach for urea‐based nitrogen fertilization, micronutrients such as manganese (Mn), and is under investigation for P (McBeath et al., 2020; Noack et al., 2010; Talboys et al., 2020).

Foliar‐applied nutrients must penetrate the leaf surface to be assimilated into plant tissues. The outermost part of the epidermal cell wall is a hydrophobic, lipid‐rich cuticle containing cutin and cutan polymers and waxes, minor phenolic and/or mineral compounds, and polysaccharides (Fernández et al., 2017; Guzmán, Fernández, García, et al., 2014). The leaf epidermis also contains structures such as stomatal pores and leaf hairs (trichomes). Hydrophilic solute penetration may preferentially occur in cuticular ledges, at the bases of trichomes, or cracks between anticlinal epidermal cells, possibly due to the presence of higher densities of intra‐cuticular molecules with polar moieties that form dynamic aqueous networks which facilitate passage (Fernández et al., 2017, 2020; Schönherr, 2006). Cells overlying leaf veins and sub‐epidermal fiber cells at the termination of the bundle sheath extension have also been linked to ion penetration, while stomata can also facilitate absorption through thin films of liquid linking the leaf surface to the interior via guard cells (Arsic et al., 2020; Bahamonde et al., 2018; Burkhardt et al., 2012; Schreel et al., 2020).

Nutrient deficiencies can alter epidermal properties and affect foliar‐applied nutrient absorption. Iron (Fe) deficiency reduced soluble cuticular lipids in peach leaves and decreased abaxial cuticle weight in pear leaves (Fernández, Eichert, et al., 2008), while P‐deficient wheat had a thinner cuticle (Fernández et al., 2014). Li et al. (2017) manipulated leaf epidermal cell wall thickness by applying methyl jasmonate (MeJa), which increased tomato leaf epidermal thickness and decreased foliar absorption of Mn, zinc (Zn), and Fe. In contrast, MeJa application thinned the epidermal cell wall in sunflower leaves and increased foliar micronutrient absorption. In another study, Zn‐deficient sunflower leaf adaxial epidermis contained more cutin and phenolics and less polysaccharides, which reduced epidermal permeability and consequently foliar‐applied Zn absorption (Li, Wang, Lombi, Wu, et al., 2018). Nutrient deficiencies also affected stomata and trichomes. P‐deficient wheat and Zn‐deficient sunflower had reduced densities of stomata and trichomes (Fernández et al., 2014; Li, Wang, Lombi, Wu, et al., 2018). Boron (B) deficient soybean had closed, collapsed and shrunken stomatal pores with smaller pore widths (Will et al., 2011). Fe‐deficient peach leaves had shorter pore lengths and lower degrees of opening (Fernández, Eichert, et al., 2008).

Latent P and Mn deficiencies are common in agricultural soils (Carstensen et al., 2019; Schmidt et al., 2013). While there have been no studies investigating at the same time the effects of Mn deficiency on leaf properties and foliar nutrient absorption, it is known that Mn deficiency in barley decreased epicuticular waxes by 40% (Hebbern et al., 2009). Despite the recent increase in studies on foliar P fertilization, mixed results have been obtained and few studies have been conducted on P‐deficient plants (Froese et al., 2020; Gross et al., 2021; McBeath et al., 2020; Talboys et al., 2020). While severely P‐deficient wheat did not absorb any foliar‐applied P, foliar‐applied P was shown to successfully restored physiological function in P‐deficient barley (Arsic et al., 2020; Fernández et al., 2014). The efficacy of foliar fertilization may also be related to the physicochemical properties of cations and anions. While cations are preferentially sorbed due to the negatively charged carboxylic groups present in plant cuticles, it is unknown whether ionic properties also affect the pathways utilized to cross the leaf epidermis (Tyree et al., 1990). This knowledge gap regarding different pathways of foliar penetration was highlighted in a recent review (Fernández et al., 2020). Few studies have investigated foliar‐applied P absorption pathways due to the difficulties in visualizing P at the relevant scale for tracing foliar penetration, which was recently addressed by utilizing vanadate (VO_4_^3−^) as a nontoxic analogous phosphate (PO_4_^3−^) ion tracer in barley leaves using LA‐ICP‐MS (Arsic et al., 2020).

The objective of this study is to characterize the effects of Mn and P nutrient deficiencies on barley leaf surface properties. Using high‐resolution LA‐ICP‐MS bioimaging, we imaged the absorption pathways of respective foliar Mn and P fertilizers (applied in concentrations reflecting agronomic practices) to nutrient deficient barley leaves. Nutrient‐specific chlorophyll *a* fluorescence assays were used to determine whether foliar‐applied Mn and P fertilizers were able to restore affected physiological processes in deficient leaves. This combination of element‐specific bioimaging techniques revealed new information regarding the combined effects of leaf surface morphology, plant nutritional status and ion properties on the foliar absorption of Mn and P, which is critical for future initiatives aiming at developing more environmentally benign fertilization practices.

## 2 MATERIALS AND METHODS

### 2.1 Plant growth conditions

Spring barley (*Hordeum vulgare* L.) cv. Irina was germinated in vermiculite for 5 days. Uniform seedlings were translocated to aerated, light‐impermeable black hydroponic units grown under controlled greenhouse conditions (Arsic et al., 2020). Each unit (4 L) contained four plants and was filled with a chelate‐buffered solution prepared in 18.2 MΩ Milli‐Q water (Milli‐Q Plus; Millipore). Three hydroponic units represented independent biological replicates. Control plants were grown with sufficient supply of all nutrients (details in Arsic et al., 2020). P‐deficient plants were grown under low P concentrations by replacing 200 μM KH_2_PO_4_ with 9 μM KH_2_PO_4_ and 200 μM KCl. Mn‐deficient plants were supplied with a starting concentration of 10 nM MnCl_2_ for the first 17 days, which was supplemented with 200 nmol MnCl_2_ per unit every second day until day 35 and increased to 400 nmol MnCl_2_ per unit (Chen et al., 2019). This was adjusted to prevent plant Mn status from declining below the threshold for “strong” Mn deficiency, defined as *F*_v_/*F*_m_ <0.5 measured by chlorophyll *a* fluorescence analysis (Schmidt, Powikrowska, et al., 2016).

### 2.2 Chlorophyll *a* fluorescence transients

Plant P and Mn status was continuously monitored by measuring the youngest fully expanded leaf (YFEL) according to the chlorophyll *a* fluorescence methods outlined in Frydenvang et al. (2015) and Schmidt, Powikrowska, et al. (2016) using a Handy PEA chlorophyll fluorometer (Hansatech Instruments). Four replicate measurements were made within each independent biological replicate unit. Hansatech leaf clips were applied to each YFEL mid‐section for at least 25 min of dark adaptation (see details in Frydenvang et al., 2015). Plant Mn status was determined by extracting the minimum (*F*_0_) and maximum (*F*_m_) fluorescence values and calculating the Photosystem II (PSII) efficiency as the ratio, *F*_v_ (*F*_m_ − *F*_0_), to *F*_m_ (Chen et al., 2019; Schmidt et al., 2013; Schmidt, Powikrowska, et al., 2016). Plant P status was related to the I step of the chlorophyll *a* fluorescence transients (OJIP transient), which were double normalized between *F*_0_ and *F*_m_ to show the relative variable fluorescence at time t:

$$V\;\left(t\right)=\frac{\text{Fluorescence}\;\left(t\right)-F_{0}}{F_{m}-F_{0}}$$

Data was extracted using PEA Plus V1.10. P status was calculated using the P‐Predict regression model (Frydenvang et al., 2015).

### 2.3 Foliar application conditions

Three types of foliar solutions were prepared: 0.2 mol L^−1^ KH_2_PO_4_ with 1 mmol L^−1^ Na_3_VO_4_ (vanadium (V) as a foliar‐P tracer (referred to hereafter as “PV”); (Arsic et al., 2020)), 20 mmol L^−1^ MnSO_4_, and a control (deionized water). P and Mn concentrations were selected to be within the realistic range typically used in agronomy while ensuring that the applied droplets did not damage leaf tissue. The vanadium concentration applied was optimized to not interfere with the membrane electrochemical gradient as verified by NPQ measurements on control and nutrient deficient plants (Arsic et al., 2020). All solutions included 0.05% (v/v) Tween‐20 and were adjusted to pH 6 using either 1 M NaOH or HCl. Foliar solutions were applied to the adaxial surface of three replicate YFELs per hydroponic treatment at tillering (21 days after sowing [DAS]) and at flag leaf emergence (45 DAS), respectively. The middle section of an attached YFEL was slid into a Petri dish lined with a moist filter paper to maintain high humidity. The part of the leaf inside the Petri dish was labeled and 30 × 5 μl droplets were applied to the leaf surface. The Petri dish lid was replaced and relative humidity rapidly increased (>95%). Droplets were left on the leaf for 6 h and remained liquid throughout the exposure period. Upon reopening the dish, drops were carefully blotted using a paper towel. Leaves were excised at the base for the following Imaging‐PAM measurements.

### 2.4 Imaging‐PAM nutrient status assays

Detached YFEL were dark‐adapted for at least 25 min before being placed inside a Walz IMAGING‐PAM (Pulse Amplitude Modulation) M‐series MAXI (Heinz Walz GmbH). Leaves were exposed to a saturating light pulse regime for diagnosing P deficiency using an NPQ assay as in Arsic et al. (2020). The *F*_v_/*F*_m_ ratio was used for leaf Mn‐status (Schmidt, Jensen, & Husted, 2016). Spatial distribution of leaf P and Mn status was analyzed using the ImagingWin v2.46i.

### 2.5 Bulk Mn and P absorption and translocation

Leaves were taken from the Imaging‐PAM and rinsed using 2 ml 2% HNO_3_ (v/v), followed by 3% ethanol (v/v) and then Milli‐Q deionized water; this was repeated three times per leaf. All rinse solutions also contained 0.05% (v/v) Tween‐20. These rinsing steps were completed after imaging as rinsing slightly dehydrated the leaves. Leaves were blotted dry then sliced into three sections, separating the middle section, which had been exposed to the foliar solutions, from the top and bottom parts of the leaf. Samples were dried at 60°C for at least 48 h before being homogenized and digested in a Milestone UltraWAVE. Samples were analyzed for total P, Mn and trace metals using a 5100 ICP‐OES or 7900 ICP‐MS (Agilent Technologies). Foliar nutrient absorption was calculated as either (P and vanadium or Mn concentration for either top, middle, or bottom treated YFEL sections [μg g^−1^ dry weight]) – (P and vanadium or Mn concentration for either top, middle, or bottom control YFEL sections [μg g^−1^ dry weight]). In this case, control leaves refer to P‐deficient and Mn‐deficient YFEL that had only deionized water and Tween‐20 applied as a foliar treatment in the middle section of the YFEL.

### 2.6 Leaf morphology and indices

Fresh YFELs were excised at the leaf base, weighed immediately to obtain fresh masses, photographed against grid‐lined paper for digital scaling and dried at 60°C for at least 48 h to obtain dry masses. Nine biological replicates were examined per hydroponic treatment and growth stage. Leaf area was analyzed using the ImageJ Fiji (Schindelin et al., 2012) and the specific leaf area (SLA) was calculated as the fresh leaf adaxial area divided by its dry mass. Leaf dry‐matter content (LDMC) was calculated as leaf dry mass divided by fresh mass. Whole plants were removed from hydroponic units, separated into shoots and roots prior to drying at 60°C (72 h) to determine root‐to‐shoot ratios (*n* = 12).

### 2.7 Leaf morphology, anatomy and adaxial surface composition

Leaf surface morphology was imaged using a FEI Quanta 200 Scanning Electron Microscope (SEM). 1 cm × 1 cm leaf pieces were cut from similar YFEL regions using a scalpel and fixed in Karnovsky's solution, for 24 h under vacuum. Following sequential dehydration in acetone, samples underwent critical point drying and sputter coating using a mixture of palladium and gold, before adaxial leaf surfaces were imaged. Stomatal and trichome densities were calculated as the number of stomata or trichomes, respectively, per 1 mm^2^ leaf area. Three biological replicates were used, with five separate regions counted within each leaf. For histological analysis, 5 mm × 20 mm leaf pieces were fixed in Karnovsky's solution for 24 h under vacuum and sequentially dehydrated in acetone prior to embedding in Spurr's resin. 2.5 μm thick sections were cut using a Leica EM UC7 Ultramicrotome prior to staining with Toluidine Blue O for 3 min. Microscopy images were captured using a Leica DM 5000B light and fluorescence microscope coupled to a Canon EOS 90D camera. Three biological replicates were analyzed per hydroponic treatment. The YFEL adaxial surface biopolymer composition was analyzed using attenuated total reflectance‐Fourier transform infrared (ATR‐FTIR) spectroscopy. For this, a Nicolet 6700 FT‐IR equipped with a Pike Technologies GladiATR diamond spectrometer (Thermo Scientific) was used. Five technical replicate measurements were made per individual leaf, with a total of three biological replicates analyzed per hydroponic treatment and growth stage, respectively. The spectral range was 4000–400 cm^−1^ with a spectral resolution of 4.0 cm^−1^ and each spectrum was based on 64 scans (128 for the background). Spectra were corrected for a creeping background baseline using a Rubberband Adaptive Baseline function (coarseness set to 15, offset adjusted until 0), and each replicate spectra was normalized using the standard normal variate in Spectragryph V1.2.14. Peak areas were calculated according to the trapz algorithm using Matlab R2014A (The Mathworks Inc.). Each peak (holocellulose, cellulose and noncellulosic structural polysaccharides (NCSPs), and waxes) had an individual linear baseline applied. Peak area ratios were calculated to determine a semiquantitative analysis of the adaxial leaf surface chemical composition by dividing the following peak areas by the peak area for holocellulose (895 cm^−1^): 1056 cm^−1^ (cellulose and NCSPs), 2850 cm^−1^ (waxes), 2918 cm^−1^ (waxes), and 3300 cm^−1^ (cellulose). Thus, the reported peak area ratios represent the apparent surface abundances of these components relative to holocellulose (Djajadi et al., 2017).

### 2.8 LA‐ICP‐MS analysis

YFEL mid‐sections were marked to represent the area contained within the Petri dish prior to dipping the leaf into foliar‐applied solutions for 15 s, before sliding the leaf into the Petri dish and incubating as described above. Whole leaves remained wet throughout the exposure period. Leaves were excised at the base and rinsed using 2% HNO_3_ (v/v), 3% ethanol (v/v) and Milli‐Q water sequentially (three times per rinse solution); all solutions also contained 0.05% (v/v) Tween‐20 (Du et al., 2015). Leaves were dried gently using a paper towel before 2 cm sections were cut using a scalpel, frozen in OCT, sectioned using a Leica CM050S cryotome and freeze‐dried (Arsic et al., 2020). Leaf sections were ablated using a nanosecond LA unit (Iridia 193 nm excimer laser ablation system, Teledyne CETAC technologies) with the following settings: Fluence: 1 J cm^−2^; scan speed: 160 μm s^−1^; repetition rate: 200 Hz; and spot size: 4 μm. A 8900 ICP‐MS (Agilent technologies) was used to obtain the element signals, operating in standard mode. Sensitivity drift was monitored before, during and after analyses using a NIST612 glass standard (NIST; National Institute for standards and technology, Gaithersburg). The monitored isotopes were either ^24^Mg, ^55^Mn, ^31^P (integration times: 0.002, 0.004, and 0.007 s, respectively), or ^24^Mg, ^31^P, and ^51^V (integration times: 0.001, 0.003, and 0.003 s, respectively). The ICP‐MS was operated with sample cone depth = 4 mm and carrier gas = 0.66 ml min^−1^. Image processing was performed in HDIP (High‐definition Image processing, Teledyne CETC technologies), including gas blank and signal drift corrections.

### 2.9 Statistical analysis

All data was analyzed using GraphPad Prism version 8.3.1 for Windows. ANOVAs were performed on ATR‐FTIR, ICP, epidermal thickness, trichome and stomatal density, and leaf indices data. Tukey's or Sidak's multiple comparisons tests were conducted to determine which groups were responsible for significant differences within the ANOVA tests. Samples were considered significantly different at *p* < 0.05 (*α*).

## 3 RESULTS

### 3.1 Effects of Mn and P deficiency on plant growth

#### 3.1.1 Whole plant growth

**FIGURE 1:**
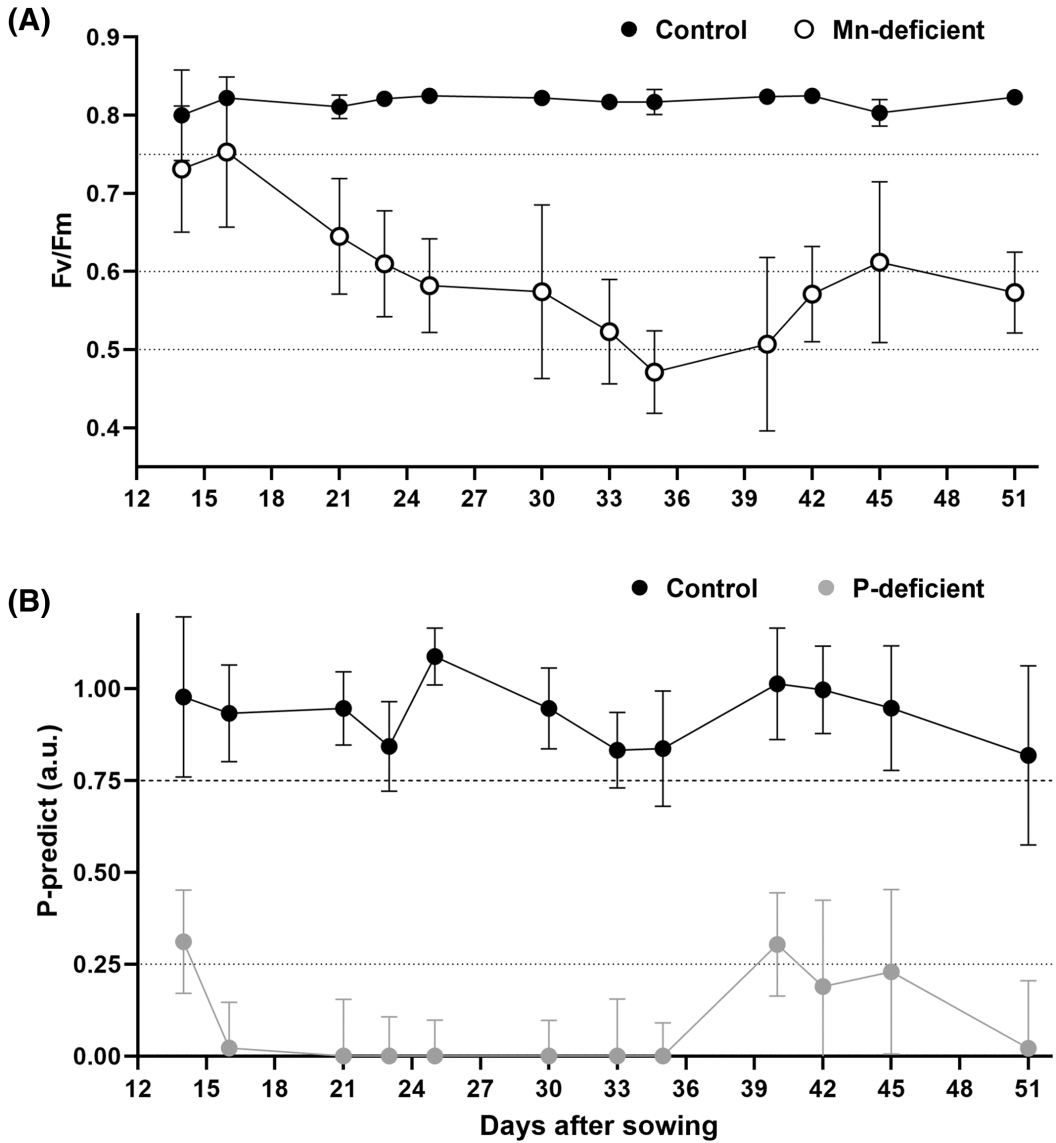
Plant nutritional status as measured by chlorophyll *a* fluorescence. (A) Mn‐status and (B) P‐status (a.u., arbitrary units). Error bars show SD (*n* = 3). See Section 2 for further details on *F*_v_/*F*_m_ and P‐predict parameters

Control plants were fully Mn‐sufficient (Figure 1A, *F*_v_/*F*_m_ values > 0.8) and P‐sufficient (Figure 1B, P‐predict > 0.75) throughout the experiment. Mn‐deficient plants declined to mild Mn deficiency at tillering (21 DAS) and further to strong deficiency on day 35 (Figure 1A).

P‐deficient plants were moderately deficient after 14 days, declining to strong deficiency by day 16, and largely remained below the threshold for strong P deficiency (<0.25), with a slight increase on day 40 (Figure 1B). YFEL elemental tissue analysis confirmed that total P concentrations in controls were above the 2000 μg g^−1^ DM threshold for P deficiency and that the total Mn concentrations were above the 15 μg g^−1^ DM threshold for Mn deficiency, at both tillering and flag leaf emergence (45 DAS) (Figure S1). P‐deficient YFELs had P concentrations below this threshold at both sampling times, as did Mn concentrations in Mn‐deficient YFELs (Figure S1).

Nutrient deficiency symptoms were latent in younger plants but became visible by flag leaf emergence. At tillering, P‐deficient plants had fewer tillers, all with red coloration (anthocyanosis) at the base, while Mn‐deficient plants had leaves that were slightly larger and paler in color compared to the control plants (Figure S2). By flag leaf emergence, P‐deficient plants were very small, with very few tillers and stiff, small, dark green leaves, while Mn‐deficient plants had a similar number of tillers compared to control plants and much larger, yellow‐green and flaccid leaves (Figure S2). Leaf indices showed that Mn and P deficiency had opposite effects on leaf morphology (Figure S3). At flag leaf emergence, Mn‐deficient YFELs had significantly higher specific leaf area (SLA) compared to controls (*p* = 0.0048), while P‐deficient YFELs had significantly lower SLA (*p* = 0.002) (Figure S3). Leaf dry matter content (LDMC) was also significantly lower in Mn‐deficient YFELs compared to control at both tillering (*p* = 0.0366) and flag leaf emergence (*p* < 0.0001), while P‐deficient YFEL LDMC did not vary significantly from the controls at either time point (Figure S3). When plants were harvested at the end of the experimental period (51 DAS), Mn‐deficient dry shoot biomass was 44% lower than control plants, while P‐deficient shoot biomass was reduced by 86% (Figure S4). Root biomass also decreased under Mn deficiency (38%) and P deficiency (58%) compared to controls, while the corresponding root: shoot ratios increased slightly under Mn deficiency (111%) and dramatically under P deficiency (306%) (Figure S4).

#### 3.1.2 Trichome and stomatal densities

**FIGURE 2:**
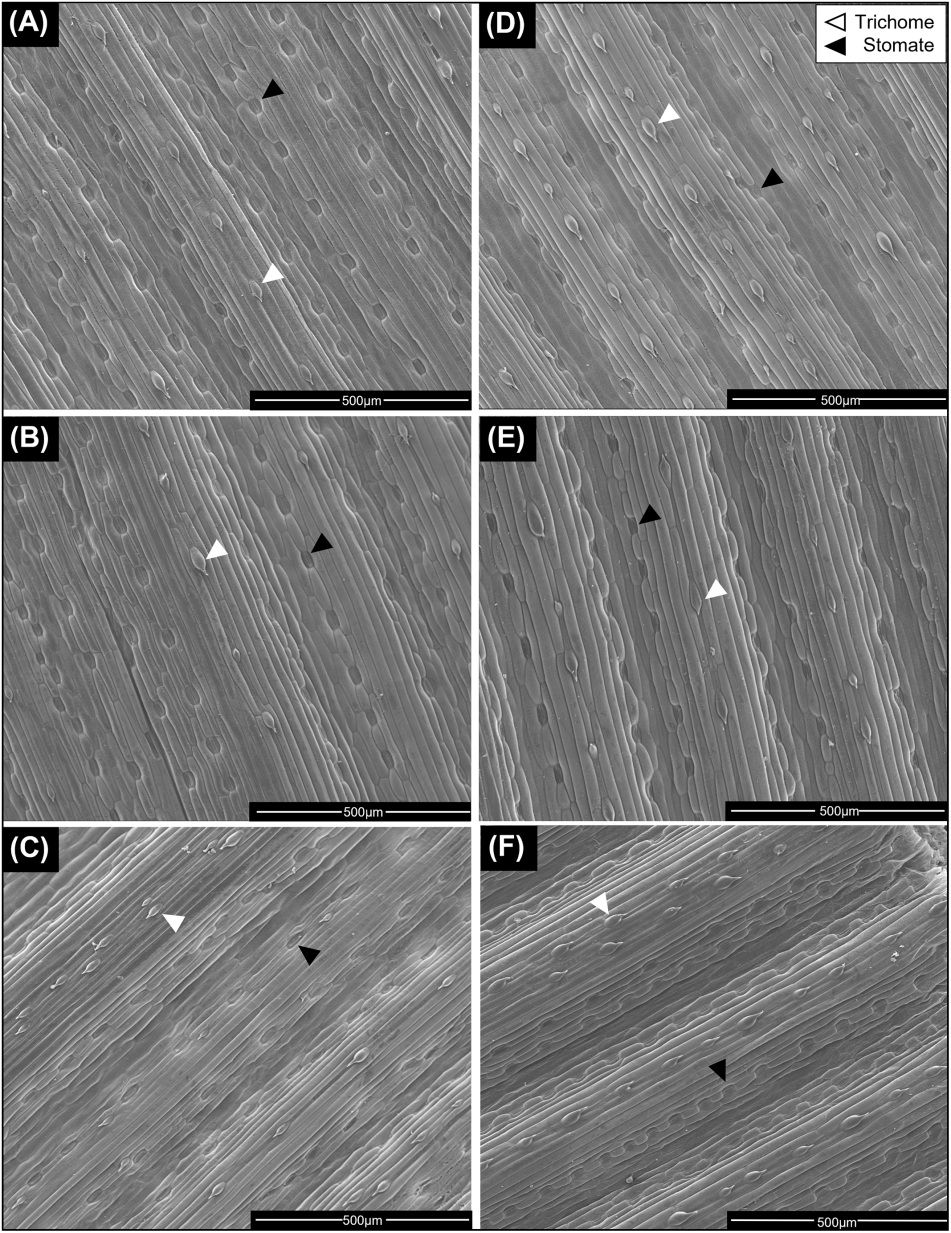
Representative SEM micrographs for the adaxial surface in barley YFEL. Figure shows plants at tillering (A) control plants, (B) P‐deficient plants, (C) Mn‐deficient plants and flag leaf emergence (D) control plants, (E) P‐deficient plants, and (F) Mn‐deficient plants. Stomatal pores (black arrows) and trichomes (white arrows) are visible in files running parallel along the leaf

The adaxial leaf surface showed epidermal cells ran in parallel files to the leaf margin, as is common in grass species. Trichomes and fiber cells were located in single files on top of vein ridges, while stomata occurred on the sides of concave “valleys” between veins (Figure 2).

**FIGURE 3:**
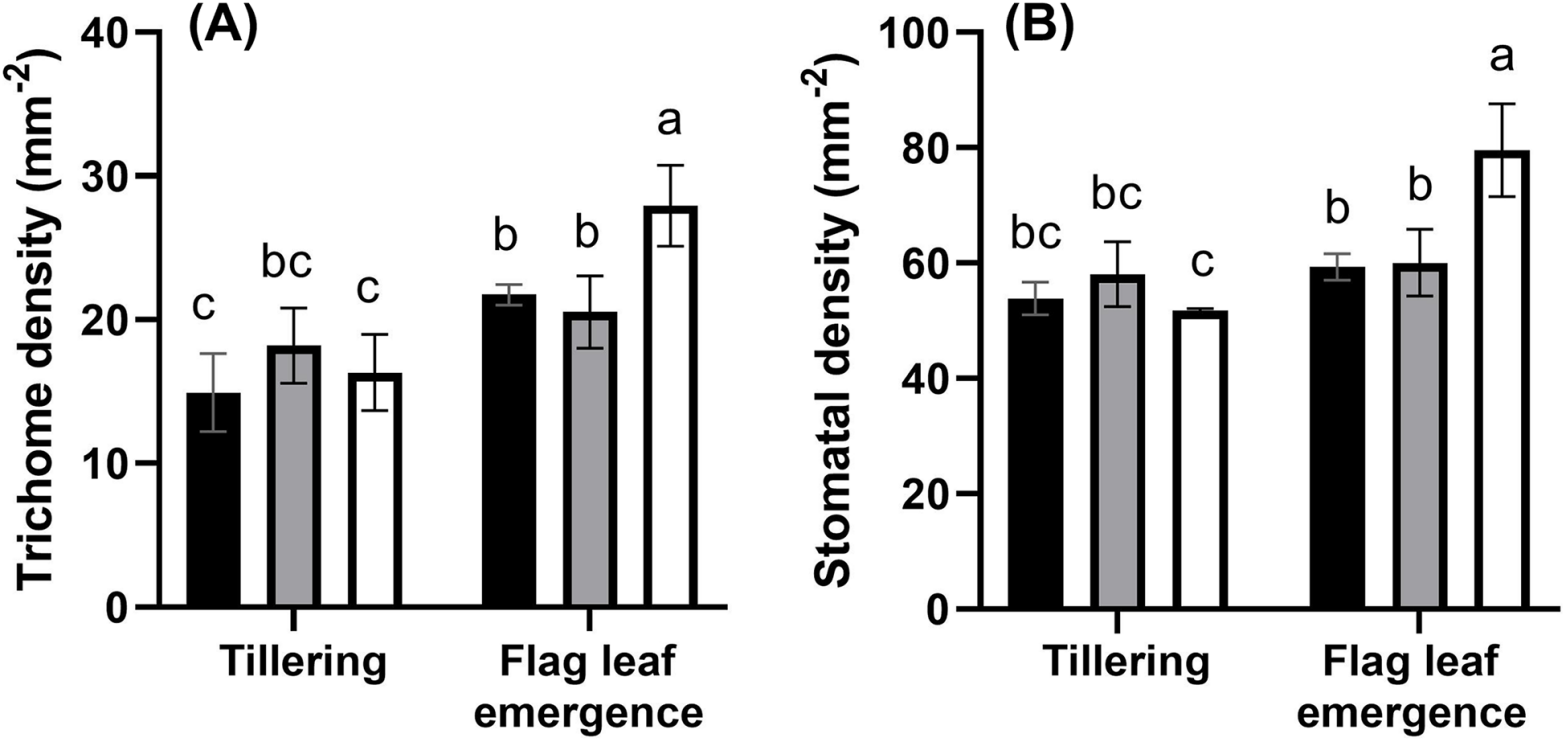
YFEL adaxial epidermis densities of (A) trichomes and (B) stomata in control, P‐deficient and Mn‐deficient plants. Error bars show SD (*n* = 3)

Trichome densities increased significantly over time in control (14.9 ± 3 mm^−2^ at tillering and 21.7 ± 4 mm^−2^ at flag leaf emergence, *p* = 0.0135) and Mn‐deficient YFELs (16.3 ± 3 mm^−2^ and 27.9 ± 5 mm^−2^, *p* = 0.0066) (Figure 3A).

Mn‐deficient YFELs also had significantly higher trichome densities compared to control and P‐deficient YFELs by flag leaf emergence (Figure 3A). P‐deficient YFELs showed no significant differences in trichome densities over time. Furthermore, Mn‐deficient YFELs showed a significant increase in stomatal density (*p* = 0.0039) between tillering (51.8 ± 5 mm^−2^) and flag leaf emergence (79.6 ± 11 mm^−2^) (Figure 3B). Mn‐deficient YFELs had significantly higher stomatal densities compared to control (59.3 ± 2 mm^−2^) and P‐deficient (60.1 ± 6 mm^−2^) YFELs at flag leaf emergence (Figure 3B). There were no significant differences in stomatal densities between treatments or over time in either control or P‐deficient YFELs (Figure 3B).

#### 3.1.3 Leaf anatomy—cuticle and epidermal cell wall thickness

**FIGURE 4:**
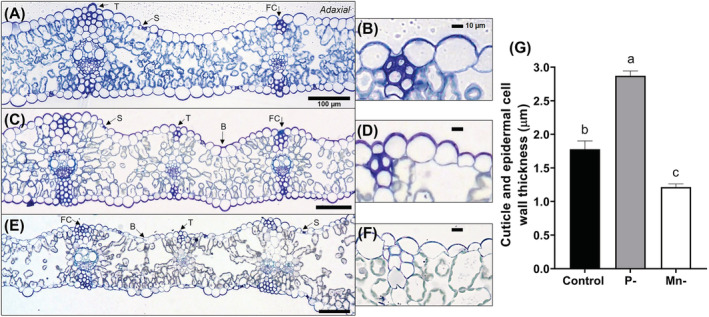
YFEL cross‐section micrographs stained with Toluidine Blue O for (A, B) control, (C, D) P‐deficient and (E, F) Mn‐deficient plants at flag leaf emergence. (G) Shows the cuticle and epidermal cell wall thickness between treatments. Error bars show SD (*n* = 3). B, bulliform cells; FC, fiber cell; S, stomate; T, trichome

Toluidine Blue O staining showed differences in leaf anatomy due to nutrient deficiencies (Figure 4).

P‐deficient YFELs had a significantly thicker cuticle (2.9 ± 0.1 μm) and slightly smaller epidermal cells compared to the control (1.8 ± 0.1 μm, *p* < 0.0001) (Figure 4C,D,G). Conversely, Mn‐deficient leaves had a significantly thinner cuticle compared to the control (1.2 ± 0.1 μm, *p* = 0.0005) that was highly altered in terms of cell size and structure (Figure 4E–G). The epidermal cells of Mn‐deficient YFEL varied greatly in size; cells flanking the vein regions were very small, flat, and elongated compared to control and P‐deficient leaves (Figure 4B,D,F). Moreover, bulliform cells were flat and folded, and fiber cells were thinner compared to the control (Figure 4E). Mn‐deficient YFELs also showed signs of structural disintegration of the mesophyll tissue (Figure 4E).

#### 3.1.4 Adaxial epidermis surface composition

**FIGURE 5:**
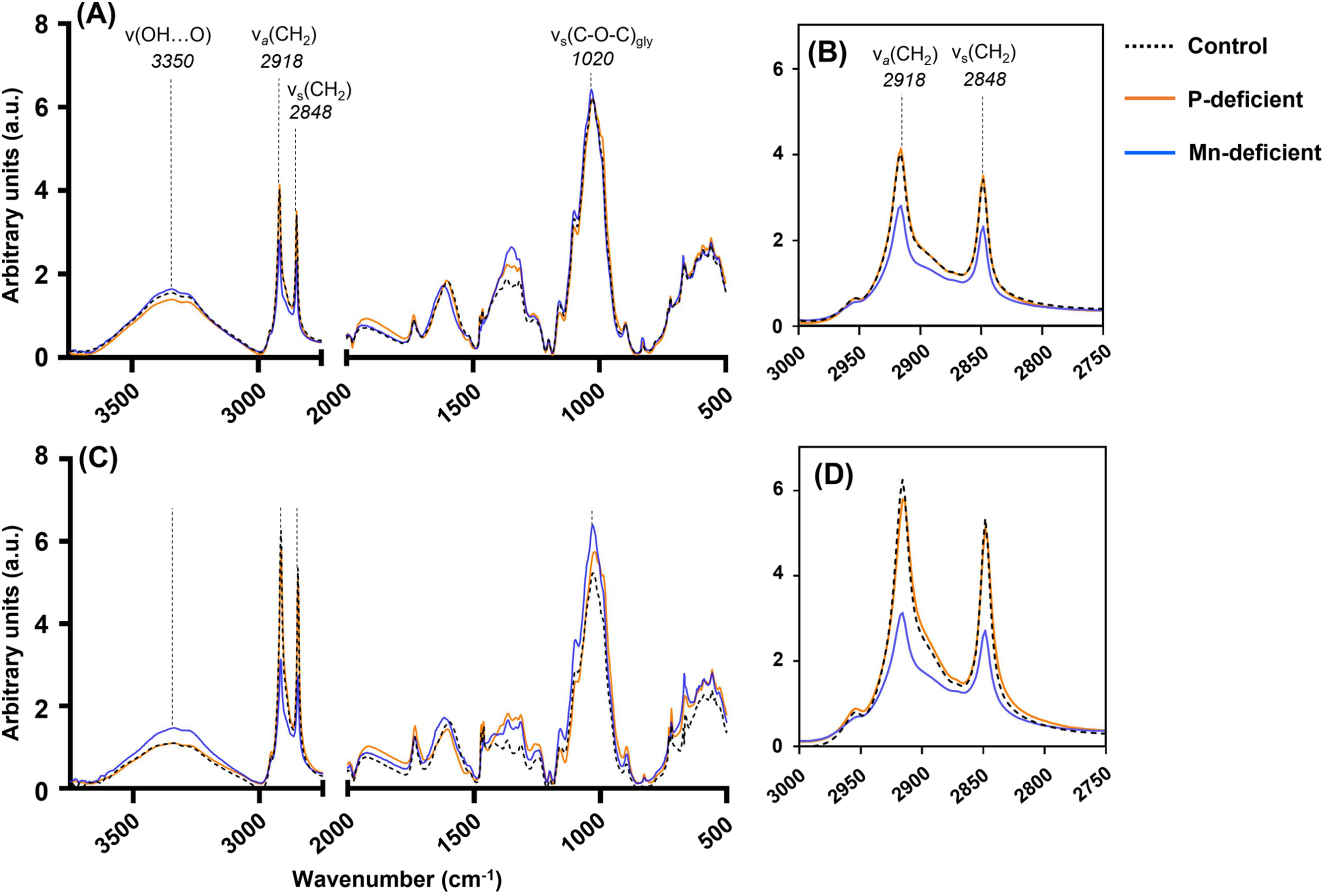
Averaged and normalized ATR‐FTIR spectra of adaxial dried YFEL surfaces at (A, B) tillering and (C, D) flag leaf emergence for control (dotted black line), P‐deficient (orange) and Mn‐deficient (blue). (B) and (D) are inset figures within the range of 3000–2750 cm^−1^ for (A) and (C), respectively

The chemical compositions of adaxial leaf surfaces differed between nutrient treatments and with plant age (Figure 5). The broad band at 3350 cm^−1^ associated with O‐H stretching has been linked to tissue hydration as moisture may bind to accessible hydroxyl groups by hydrogen bonding (Lupoi et al., 2015). The glycosidic bond at 1020 cm^−1^ is likely associated with cellulose and noncellulosic structural polysaccharides (NCSPs) (Durak & Depciuch, 2020; Lupoi et al., 2015). Peak area ratios showed P‐deficient YFEL had significantly lower absorbance in these regions, especially at flag leaf emergence, compared to both younger P‐deficient YFEL and other nutrient treatments (Figure S5A,B). Mn‐deficient YFEL had significantly higher absorbance in the 1020–1056 cm^−1^ range compared to the control at flag leaf emergence (Figure S5B). The normalized spectra showed differences in absorbance in the “main lipid region” between 2800 and 3000 cm^−1^, specifically the CH_2_ asymmetric band (2945–2866 cm^−1^) and the CH_2_ symmetric band (2866–2820 cm^−1^) (Willick et al., 2018) (Figure 5B,D). This region is associated with long‐chain aliphatic compounds largely linked to the majority of cutan, cutin, and waxes (Heredia‐Guerrero et al., 2014). Control and P‐deficient YFEL had similar absorbances, which increased in both treatments by flag leaf emergence (Figure 5B,D). Peak area ratios showed Mn‐deficient YFEL had significantly lower absorbance in both regions compared to control YFEL at flag leaf emergence (Figure S6A,B).

### 3.2 Foliar Mn applications

#### 3.2.1 Foliar‐applied Mn accumulated in epidermal cells and mesophyll tissue

**FIGURE 6:**
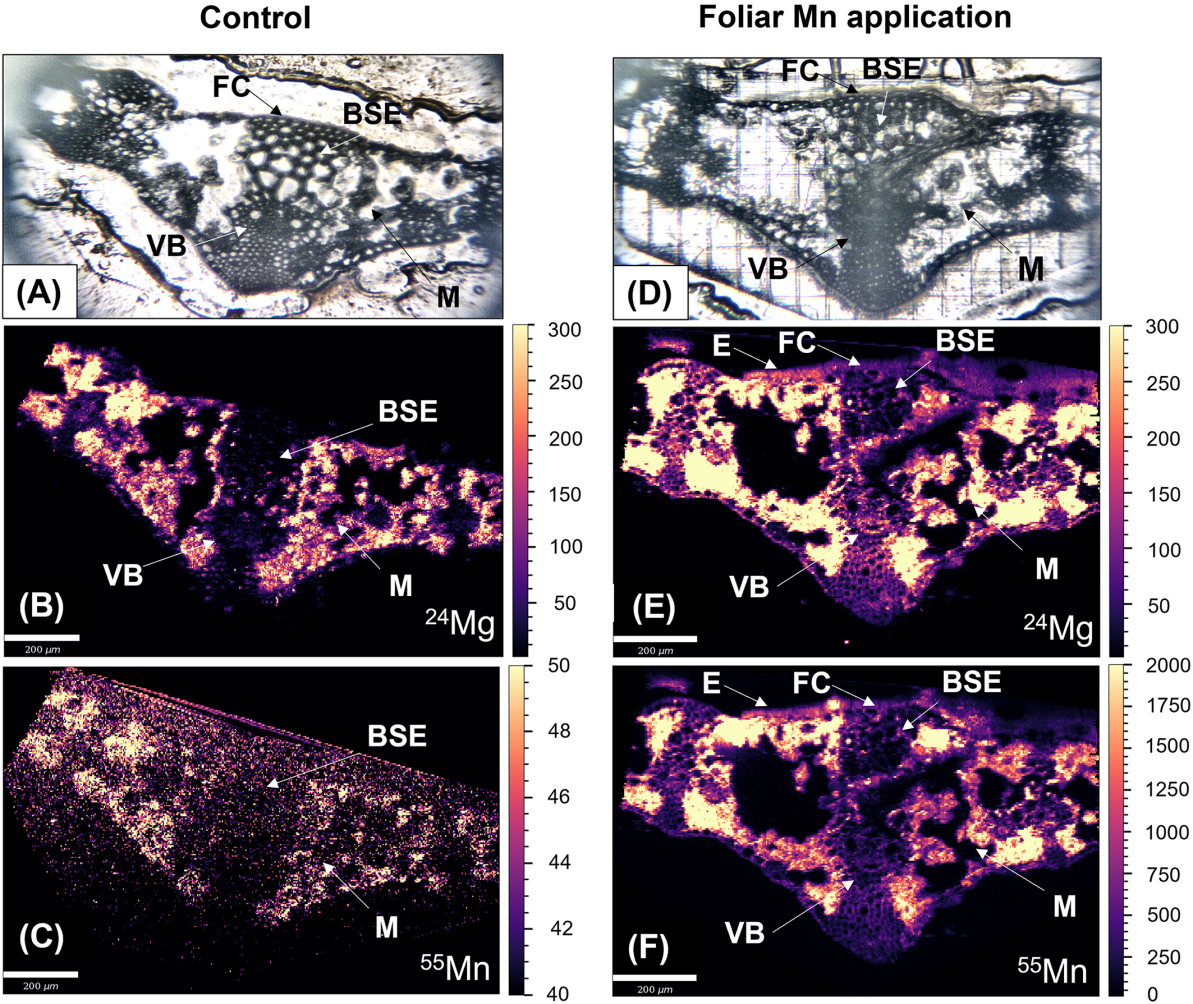
Representative LA‐ICP‐MS scans of Mn‐deficient leaf midrib cross‐sections at flag leaf emergence. (A, D) Bright field microscopy images, (B, E) show the ^24^Mg elemental distributions and (C, F) show ^55^Mn element distributions. White scale bars indicate 200 μm for microscopy images. Element scale bars indicate counts (signal intensity) (NB: 0–50 counts for the control, 0–2000 counts for the foliar Mn treatment). BSE, bundle sheath extension; E, epidermis; FC, fiber cell; M, mesophyll; VB, vascular bundle

Control leaf cross‐sections showed that native Mn distributions were associated with mesophyll tissue, with little to no Mn in vascular bundles, bundle sheath extensions or in the epidermis (Figure 6C).

Foliar‐applied Mn leaf cross‐sections showed signal intensity was still highest in the mesophyll (Figure 6F). Furthermore, Mn hotspots were visible in some epidermal cells immediately above the mesophyll tissue (Figure 6F). Similar Mn distributions were observed in leaf cross‐sections of lower‐order vascular bundles (Figure S7).

Bulk Mn concentrations in the treated part of the leaf were compared, as there was no detectable translocation of Mn above or below the zone of foliar application within 6 h (Figure S8). Following foliar Mn application, all treatments had high Mn concentrations (>150 μg Mn g^−1^ DW) (Figure S9). Mn‐deficient YFEL had significantly higher Mn concentrations compared to Mn‐sufficient YFEL in plants at tillering (interaction between plant Mn status and growth stage, *p* = 0.0246), but not at flag leaf emergence (Figure S9). This was likely due to a dilution effect, as Mn‐deficient YFEL at flag leaf emergence were significantly larger and had higher fresh weights than control YFEL.

#### 3.2.2 Foliar‐applied Mn rapidly restored functionality in Mn‐deficient YFEL

**FIGURE 7:**
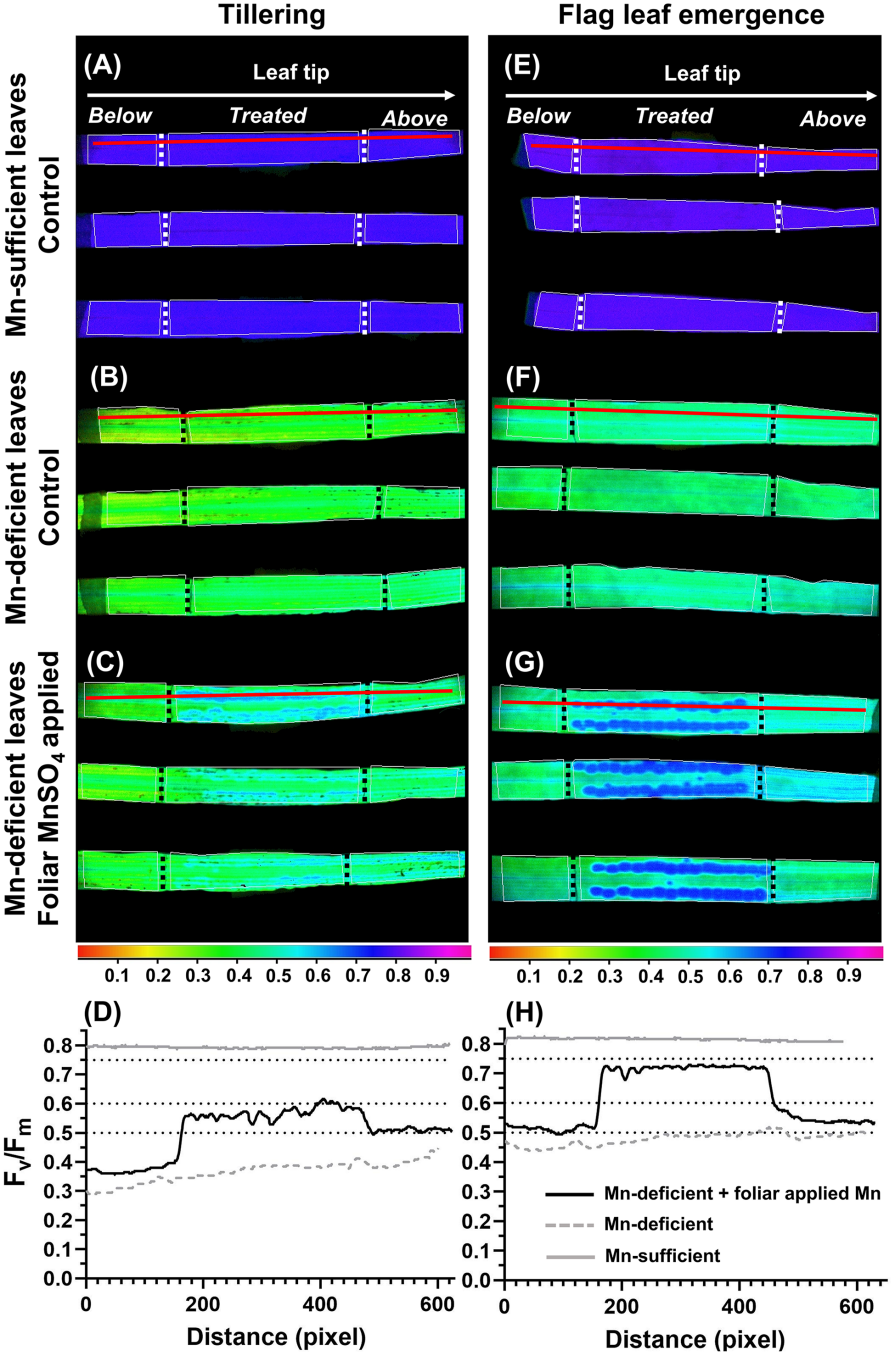
*F*_v_/*F*_m_ imaging‐PAM assay of physiological plant Mn status in YFEL at tillering and flag leaf emergence. (A, E) Control Mn‐sufficient, (B, F) Mn‐deficient plants, and (C, G) foliar Mn applications to Mn‐deficient plants. Colored scale bar shows *F*_v_/*F*_m_ values from 0 to 1. Vertical dotted lines indicate zone of foliar application. (D, H) Transects (red line) were measured through a zone of foliar‐applied droplets parallel to the leaf margin, from below to above the treated leaf region, in Mn‐deficient leaves with foliar Mn applications (black line), control Mn‐sufficient leaves (gray line), and control Mn‐deficient leaves (gray dashed line). Horizontal dotted lines indicate mild (0.75–0.6), moderate (0.6–0.5) or severe (<0.5) Mn deficiency

Transects showed that foliar Mn applications in YFEL at flag leaf emergence almost fully restored Mn functionality in areas below the droplet application (Figure 7).

Foliar Mn applications on YFEL at tillering increased *F*_v_/*F*_m_ values from severe to moderate Mn deficiency (Figure 7D). The imaging‐PAM confirmed the Mn status of Mn‐sufficient and Mn‐deficient controls (Figure 7A,E). Transects measured in replicate leaves showed similar results (Figure S10). No adverse effects were measured in Mn‐sufficient leaves that received foliar‐applied Mn (Figure S11).

### 3.3 Foliar P applications

Foliar‐applied P solutions contained 1 mmol L^−1^ vanadate (Na_3_VO_4_), which previously was shown to be a useful and appropriate analogous tracer for P using LA‐ICP‐MS, without showing negative effects on leaf transpiration, respiration or stomatal conductance at the applied concentration (Arsic et al., 2020). This solution is referred to as a “PV” solution in the text (see Section 2).

#### 3.3.1 P and vanadate accumulated in trichomes and fiber cells

**FIGURE 8:**
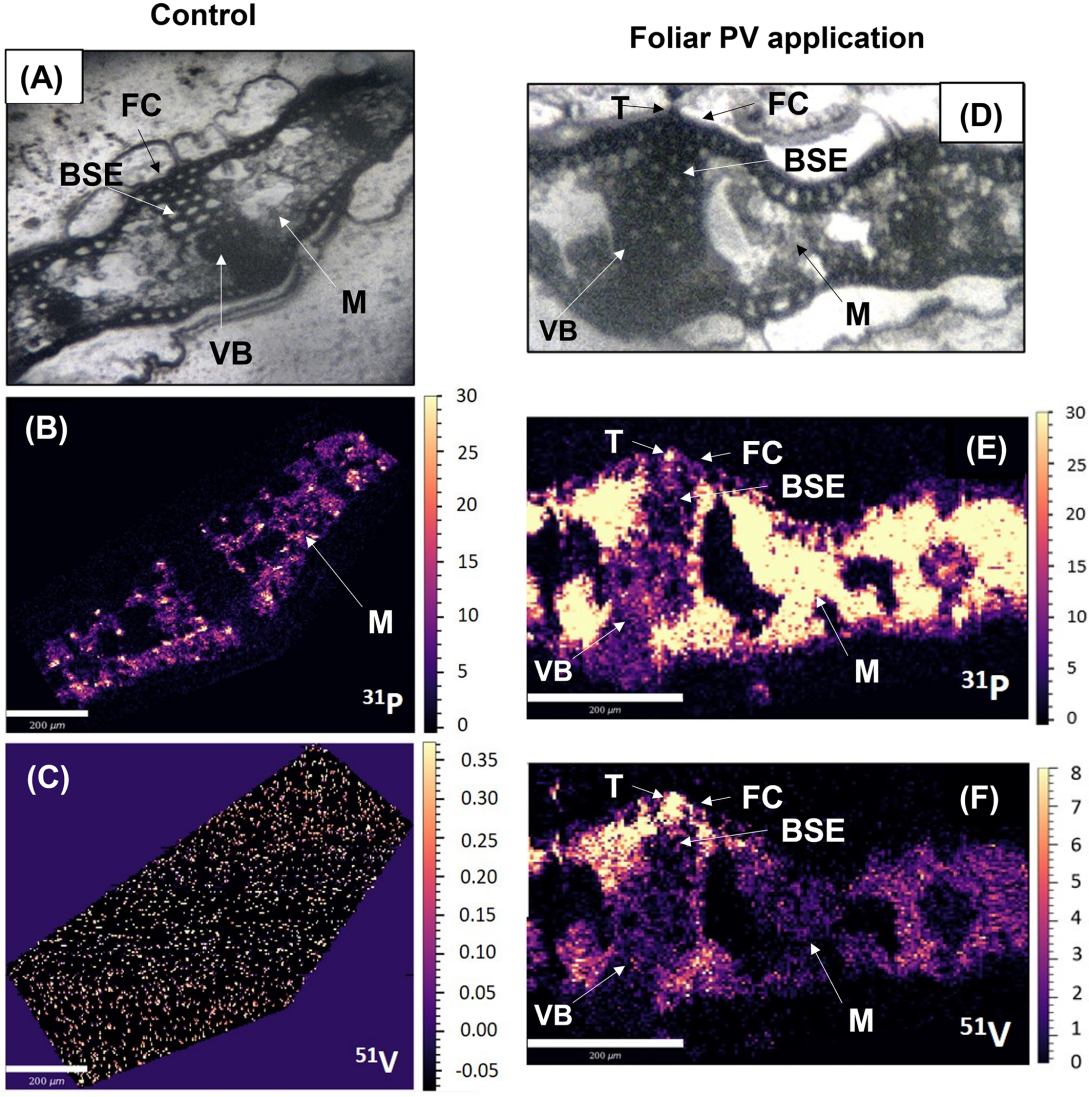
Representative LA‐ICP‐MS scans of P‐deficient leaf cross‐sections at flag leaf emergence, where (A, D) are the bright field microscopy images, (B, E) show the ^31^P elemental distributions and (C, F) show ^51^V elemental distributions. White scale bars indicate 200 μm for microscopy images. Elemental scale bars indicate counts (signal intensity) (NB: 0–0.35 counts (^51^V) for the control, 0–8 counts (^51^V) for the foliar PV treatment). BSE, bundle sheath extension; FC, fiber cell; M, mesophyll; T, trichome; VB, vascular bundle

Control leaf cross‐sections showed P distributions were associated with the mesophyll, with little to no counts visible in the vascular bundle, bundle sheath extension or the fiber cells (Figure 8A,B).

**FIGURE 9:**
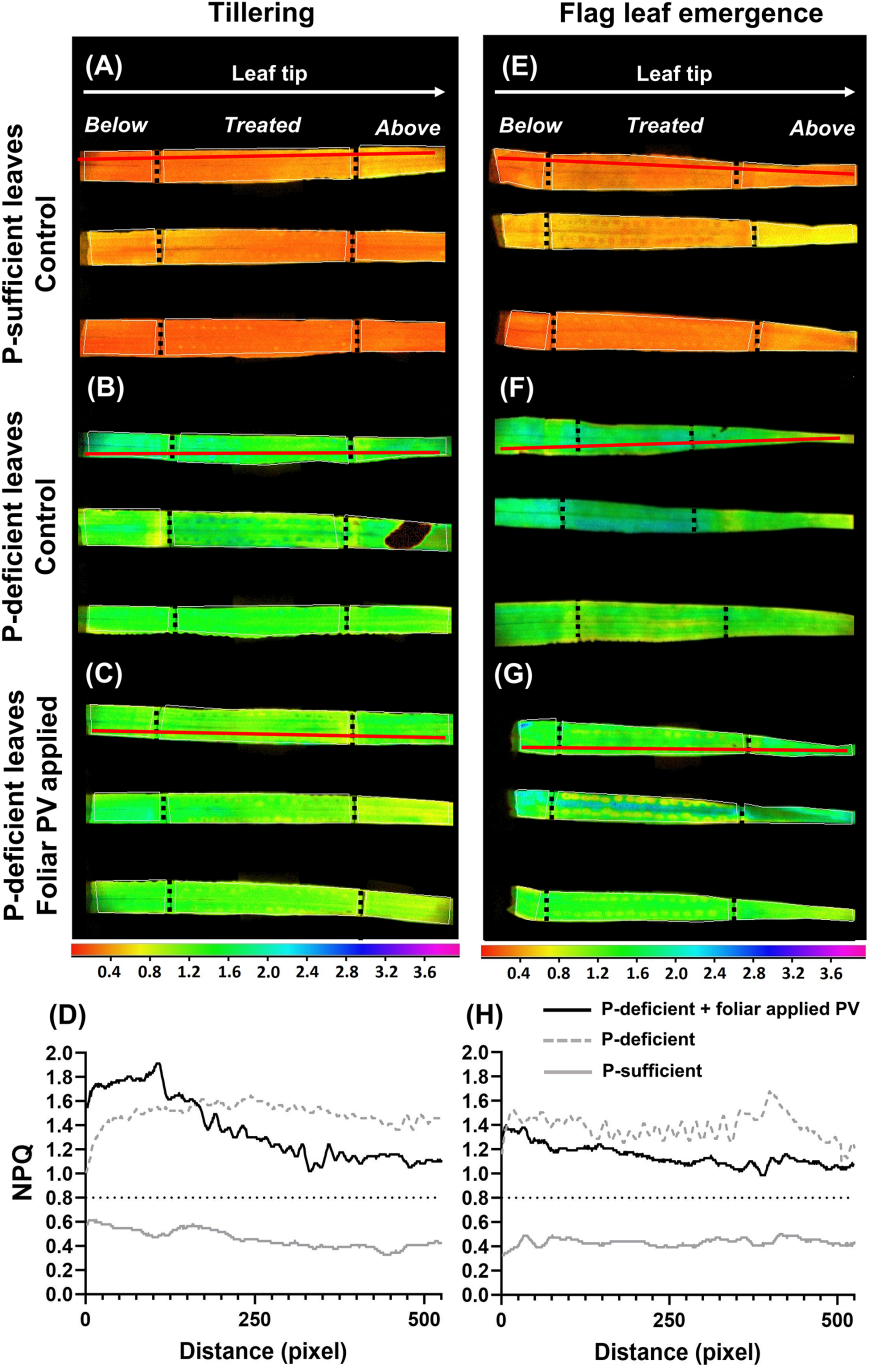
NPQ imaging‐PAM assay of physiological plant P status YFEL at tillering and flag leaf emergence in (A, E) P‐sufficient (control), (B, F) P‐deficient plants, and (C, G) foliar PV applications to P‐deficient plants. Colored scale bar shows NPQ values from 0 to 4. Vertical dotted lines indicate zone of foliar application. (D, H) Transects (red line) were measured through a zone of foliar‐applied droplets parallel to the leaf margin, from below to above the treated leaf region, in P‐deficient leaves with foliar PV applications (black line), control P‐sufficient leaves (gray line) and control P‐deficient leaves (gray dashed line). The horizontal dotted line indicates the approximate threshold for P deficiency (>0.8)

There was almost no V present in the control leaf cross‐section, confirming its usefulness as a tracer in plant tissue (Figure 8C). Following foliar PV applications, P signal intensities increased throughout the leaf cross‐section and were highest in the mesophyll tissue, with a small hotspot identifiable in an adaxial trichome (Figure 8E). V signal intensities were highest in the trichome, fiber cells, bundle sheath extension cells and the adjacent mesophyll cells immediately under the adaxial epidermis (Figure 9F). Replicate scans showed similar elemental distributions, with V hotspots in fiber cells located at the leaf tip (Figure S12) and in adaxial epidermal cells (likely containing fiber cells) located above lower order vascular bundles (Figure S13).

As V was used as a P tracer, total leaf V concentrations were measured. V concentrations in the treated part of the leaf were compared as there was no apparent translocation above or below the zone of foliar application within 6 h (Figure S14). Following foliar PV applications, V leaf concentrations increased in both P‐sufficient and deficient leaves at both growth stages (Figure S15). No significant differences were detected between treatments.

#### 3.3.2 Foliar‐applied P did not restore functionality in P‐deficient YFEL

Following foliar P applications, there was no indication of physiological restoration at either growth stage (Figure 9C,G).

Transects showed a slight decrease in NPQ values after foliar PV applications compared to P‐deficient controls. However, this did not reach approximate P‐sufficient NPQ values (Figure 9D,H). Transects measured in replicate leaves showed similar results (Figure S16). P‐deficient controls were fully P‐deficient (>0.8) (Figure 9B,F). P‐sufficient controls remained P‐sufficient (<0.8) whether deionized water or foliar PV solutions were applied (positive controls) (Figures 9C,D; and S17).

## 4 DISCUSSION

Foliar‐applied Mn restored nutrient functionality in Mn‐deficiency YFEL below applied droplets, while foliar‐applied P failed to do so in P‐deficient YFEL. While there were few differences in adaxial leaf surface morphology and chemical composition between control and Mn‐deficient YFEL at tillering, foliar‐applied Mn was able to restore PSII functionality at both Mn‐deficient growth stages. This indicates that adaxial leaf surfaces are largely permeable to foliar‐applied Mn, likely due to the ionic properties of the Mn^2+^ ion (see Section 4.4 for further discussion). Mn deficiency resulted in multiple modified adaxial leaf properties at flag leaf emergence that increased permeability to foliar‐applied Mn, while P deficiency led to modifications that made P sorption more difficult. When comparing Mn and P absorption, factors such as different ionic properties, concentration gradients across the leaf surface, and nutrient demand must also be considered.

### 4.1 Nutrient deficiencies modified cuticle and epidermal cell wall properties

The cuticle, stomata, trichomes, veins, and fiber cells can facilitate foliar solute absorption (Fernández et al., 2020). The cuticle is a highly heterogeneous lipid‐rich, outermost region of the epidermal cell wall, containing a gradient of hydrophobic to hydrophilic compounds from the outer to inner epidermal cell wall surface, although polysaccharides have been shown to be heterogeneously distributed throughout (Fernández et al., 2017). Polysaccharides form intrusive hydrophilic networks connected to the underlying epidermal cell wall matrix, where in theory water sorbs to molecular polar moieties and forms transcuticular connections facilitating ion penetration (Fernández et al., 2020).

Mn‐deficient YFELs had a thinner cuticle and epidermal cell wall, with lower abundances of waxes and higher abundances of polysaccharides compared to controls at flag leaf emergence (Figures 4 and 5). As Mn is essential for the Oxygen Evolving Complex in PSII, Mn deficiency degraded PSII complex cores, leading to thylakoid disintegration and mesophyll disorganization (Figure 4) (Bricker et al., 2012; Schmidt, Jensen, & Husted, 2016). Mn deficiency was shown to reduce leaf thickness and the cuticular wax layer (Alejandro et al., 2017; Hebbern et al., 2009; Papadakis et al., 2007). Conversely, P‐deficient YFELs had a thicker cuticle and epidermal cell wall, with lower abundances of polysaccharides compared to controls (Figures 4 and 5). As P deficiency decreased cuticle thickness in wheat, responses may vary between plant species (Fernández et al., 2014). P‐deficient plants became more resource conservative (e.g. fewer leaves with lower SLA; Figure S3). A thicker and less hydrated epidermal cell wall, with lower relative abundances of polysaccharides, could increase leaf longevity via protection from excess water loss, pathogenic infection, or photodamage (Yeats & Rose, 2013).

While other studies have used physical, chemical and/or enzymatic techniques to isolate and study the physicochemical composition of the epidermis or cuticle (Fernández et al., 2017; Li, Wang, Lombi, Wu, et al., 2018; Schreiber, 2005), ATR‐FTIR was conducted on whole leaves in this study as epidermal damage was observed, even after careful attempts at physical separation, while extensive enzymatic dissolution protocols were unable to fully isolate the cuticle. Furthermore, the cuticle is being recognized as a lipidized region of the epidermal cell wall that also contains polysaccharides; hence chemical extraction methods may introduce artifacts (Fernández et al., 2017, 2020; Guzmán, Fernández, Graça, et al., 2014). Thus, lower abundances of waxes in Mn‐deficient YFEL could simply be due to a thinner epidermal cell wall, as isolated cuticles were not measured, and the ATR‐FTIR penetration depth varies between compounds (Djajadi et al., 2017). Further studies should investigate possible compositional differences using quantitative techniques (e.g. gas chromatography mass spectrometry).

### 4.2 Mn deficiency, but not P deficiency, increased trichome and stomatal densities at flag leaf emergence

Foliar solute penetration is affected by stomatal densities and aperture widths (Eichert et al., 1998; Eichert & Goldbach, 2008; Schönherr & Bukovac, 1972). Stomatal foliar absorption occurs via a thin liquid film lining the guard cell surface and connects the leaf surface and interior, where the increased wettability of the guard cell is “activated” via external processes, including the presence of hygroscopic particles, epistomatal mucilage, fungal hyphae, or bacteria (Burgess & Dawson, 2004; Burkhardt et al., 2012; Eichert et al., 2008; Westhoff et al., 2009). More recently, trichomes have been shown to be important for foliar‐applied solute penetration (Li, Wang, Lombi, Cheng, et al., 2018; Li, Wang, Lombi, Wu, et al., 2018; Li et al., 2019, 2021). However, this varies between species and depends on trichome hydrophilicity, glandular properties, and cellular structure (Fernández et al., 2011; Li, Wang, Lombi, Cheng, et al., 2018; Xie et al., 2020).

Mn‐deficient YFEL had higher densities of adaxial stomata and trichomes compared to controls at flag leaf emergence, which could increase the permeability of the epidermis, facilitating Mn absorption (Figure 3). While P deficiency reduced trichome and stomatal densities in wheat (Fernández et al., 2014), there were no significant differences in densities in this study between P‐deficient and control YFEL at either growth stage (Figure 3). However, LA‐ICP‐MS scans showed hotspots of vanadate absorption in trichomes and fiber cells, indicating these cell types could be more permeable than epidermal pavement cells (Figure 9). While vanadate has been shown to be an appropriate tracer for identifying these leaf surface structures associated with foliar‐applied P, comparing elemental distributions inside tissues should be made with caution as vanadium speciation may vary (see Arsic et al., 2020). Leaf veins and fiber cells had increased permeability to foliar‐applied CaCl_2_, and phosphate and vanadate (Arsic et al., 2020; Bahamonde et al., 2018). Beech leaf trichomes had a pectin‐rich, cuticle‐free cell wall layer that increased absorption and redistribution of foliar‐applied silver (Ag) ionic and fluorescent tracers, whereby sub‐epidermal fiber cells facilitated translocation into the leaf tissue via the cell lumen apoplast (Schreel et al., 2020). Leaf hairs absorbed foliar‐applied water in xerophilous leaves due to externally located pectin in their cell walls, while nonglandular trichomes in sunflower absorbed foliar‐applied Zn via hydrophilic trichome bases (Li et al., 2021; Losada et al., 2021). Further studies should investigate the composition of barley trichomes and fiber cells to better understand these processes.

### 4.3 Restoration of nutrient functionality

Foliar‐applied Mn was able to restore nutrient functionality at both tillering and flag leaf emergence (Figure 7). The higher *F*_v_/*F*_m_ values reported at flag leaf emergence indicate that the resulting multiple modifications to adaxial YFEL surface morphology (increased stomatal and trichome densities) and chemical composition increased permeability to foliar‐applied Mn. Further research should be conducted to determine the effect of single variables (e.g. increased stomatal densities) on foliar‐applied Mn absorption.

Unlike Mn, foliar‐applied P did not restore nutrient functionality in P‐deficient leaves (Figures 6 and 8). LA‐ICP‐MS scans showed that some foliar uptake occurred due to vanadate signal intensities in trichomes, fiber cells, bundle sheath extension cells and the mesophyll (Figure 9). It is unlikely that rapid translocation away from the treated leaf region prevented enough foliar‐applied P from accumulating to restore P functionality given the short application time. Furthermore, a similar study found 65% of the absorbed foliar‐applied P in P‐deficient wheat remained in the treated region of the treated YFEL even after 48 h (Fernández et al., 2014). As a previous study under similarly high humidity showed restoration of P functionality in P‐deficient barley after 24 h, it is possible that a 6 h application period was rather too short to allow sufficient P penetration to restore physiological function (Arsic et al., 2020). Future studies should investigate how foliar application periods affect P absorption and accumulation in treated leaves in relation to the translocation of foliar‐applied P to the rest of the plant (e.g. using radioisotope labeling). P is an essential plant macronutrient where even short‐term deficiency leads to a range of impaired physiological processes and adaptations (Carstensen et al., 2019; de Bang et al., 2020). The reduced availability of orthophosphate in the chloroplast stroma under P‐deficient conditions inhibits ATP synthase activity and ultimately limits photosynthesis through a series of reactions in the electron transport chain (Carstensen et al., 2018).

### 4.4 Multiple factors affect foliar solute penetration

The concentration gradient across the leaf surface drives foliar absorption via diffusion. Cytoplasmic concentrations vary enormously between P (1–5 mmol L^−1^) and Mn (5 × 10^−3^ mmol L^−1^) (Clarkson, 1988; Lee et al., 1990; Lee & Ratcliffe, 1993). Here, the ratio between foliar‐applied Mn to cytoplasmic Mn concentrations (4000‐fold) was approximately 100 times higher than the ratio between foliar‐applied P and cytoplasmic P concentrations (40‐fold), which would comparatively favor Mn absorption. The physiological nutrient demand differences are also large. P is a macronutrient, so higher internal concentrations are required compared to micronutrient Mn. The amount of absorbed foliar‐applied Mn vs P solution needed to raise tissue concentrations to the “sufficient” nutrient threshold in the area just below the 5 μl droplet was estimated, assuming no translocation occurred (Table S1). 80‐times more foliar‐applied P than Mn would need to be absorbed to meet the threshold for respective nutrient sufficiency.

Cuticle properties also affect solute absorption. The ionic radius of H_2_PO_4_^−^ ions (223 pm) is much larger than Mn^2+^ (83 pm); hydrated radiuses would be larger still (Simoes et al., 2017). Reported putative aqueous pore radiuses also vary. Indirect calculations range between 0.3 and 0.45 nm (Popp et al., 2005; Schönherr & Schmidt, 1979), 2.0 and 2.12 nm (Beyer et al., 2005; Eichert & Goldbach, 2008), and up to 43 nm in the periphery of stomatal guard cells (Eichert et al., 2008). Additionally, as cuticles are polyelectrolytes with isoelectric points around 3, they are negatively charged and selectively permeable to cations over anions above this pH (Schönherr & Huber, 1977). Cell walls are negatively charged due to the presence of carboxylic acid functional groups within pectins, which would limit PO_4_^3−^ absorption over Mn^2+^ (Shomer et al., 2003). Thus, effective cuticular penetration could be the reason why foliar‐applied Mn was observed in epidermal cells and the mesophyll in this study, while P and vanadate appeared more limited to trichomes and fiber cells (Figures 7 and 9).

## 5 CONCLUSION

The unique combination of high‐resolution bioimaging techniques revealed a link between the absorption pathways of respective foliar‐applied Mn and P ions and the restoration of physiological processes in nutrient deficient leaves. Foliar‐applied Mn absorption was facilitated by a thinner cuticle and epidermal cell wall with higher abundances of polysaccharides but lower abundances of waxes at flag leaf emergence, which enabled a fast restoration of photosynthetic processes within 6 h. Within the same time frame (6 h), foliar P applications were not able to restore functionality in P‐deficient leaves, likely due to the combination of a thicker cuticle and epidermal cell wall with lower abundances of polysaccharides leading to lower P penetration of leaf barriers, and a higher relative nutrient demand for P. Trichomes and fiber cells constitute an important pathway for foliar P penetration, while Mn accumulated in epidermal cells and in the photosynthetically active mesophyll. In addition, higher nutrient demand, lower P concentration gradient across the leaf surface, ion radius, and ionic charge are also likely parameters limiting foliar‐applied P absorption relative to Mn. Foliar nutrient absorption is a dynamic process affected by plant nutrient status, leaf surface structure and composition, and not least the physicochemical properties of the mineral ions applied. Understanding these dynamics is vital to designing effective foliar fertilizers for crop nutrition with the lowest possible environmental impacts.

## AUTHOR CONTRIBUTIONS

Søren Husted, Jan K. Schjoerring, Daniel P. Persson, Enzo Lombi, Casey L. Doolette, and Maja Arsic contributed to study conception and/or experimental design; Maja Arsic conducted plant, microscopy, foliar applications, chlorophyll fluorescence, and ICP preparation and analysis; Daniel P. Persson and Maja Arsic conducted LA‐ICP‐MS experiments and analysis; Lisbeth G. Thygesen and Maja Arsic planned, conducted and analyzed ATR‐FTIR experiments; Maja Arsic, Søren Husted, and Jan K. Schjoerring drafted the paper; all authors have read, corrected and approved the final version.

## Supporting information


**Figure S1** Leaf concentrations of (a) phosphorus and (b) manganese. Black bars show control leaves (P‐ and Mn‐sufficient), gray bars show P‐deficient leaves and white bars show Mn‐deficient leaves. Dotted lines indicate published thresholds for P‐deficiency (2000 μg g^−1^ DM) and Mn‐deficiency (15 μg g^−1^ DM). Error bars show SD (*n* = 3).
**Figure S2** Control (P‐sufficient and Mn‐sufficient), P‐deficient and Mn‐deficient barley plants at tillering (21 DAS) and following flag leaf emergence (51 DAS).
**Figure S3** YFEL leaf indices by nutrient deficiency and growth stage at tillering (T) or flag leaf emergence (FLE). (a) Specific leaf area and (b) leaf dry matter content for control (black bars), P‐deficient (gray bars) and Mn‐deficient (white bars) plants. Error bars show SD (*n* = 9).
**Figure S4** Total dry biomass production for control (black bars), P‐deficient (gray bars) and Mn‐deficient plants (white bars) at 51 DAS. (a) Shoot weight; (b) root weight; (c) root: shoot ratio. Error bars show SD (*n* = 6).
**Figure S5** ATR‐FTIR peak area ratio of wavenumbers representing (a) cellulose (3300–3350 cm^−1^) and (b) cellulose and noncellulosic structural polysaccharides (NCSPs) (1020–1056 cm^−1^), each relative to the peak area of holocellulose (895 cm^−1^) for control, P‐deficient and Mn‐deficient YFEL adaxial scans. Black bars represent YFEL at tillering, gray bars represent YFEL at flag leaf emergence. Data points show the mean and error bars show SD (*n* = 3). Different letters indicate statistically significant differences between means (ANOVA, *α* = 0.05).
**Figure S6** ATR‐FTIR peak area ratio of wavenumbers representing the (a) CH_2_ asymmetric band (2945–2866 cm^−1^) and (b) CH_2_ symmetric band (2866–2820 cm^−1^), each relative to the peak area of holocellulose (895 cm^−1^) for control, P‐deficient and Mn‐deficient YFEL adaxial scans. Black bars represent YFEL at tillering, gray bars represent YFEL at flag leaf emergence. Data points show the mean and error bars show SD (*n* = 3). Asterisks indicate statistically significant differences between means (ANOVA, *α* = 0.05).
**Figure S7** Representative LA‐ICP‐MS scans of Mn‐deficient YFEL cross‐sections at flag leaf emergence, where (a, d) are the bright field microscopy images, (b, e) show the ^24^Mg elemental distributions and (c, f) show ^55^Mn elemental distributions. White scale bars indicate 200 μm for microscopy images. Element scale bars indicate counts (signal intensity) (NB: 0–36 counts for the control, 0–2000 counts for the foliar Mn treatment). BSE = bundle sheath extension, FC = fiber cell, M = mesophyll, VB = vascular bundle.
**Figure S8** Leaf Mn concentrations measured by ICP‐MS in leaf regions below, containing and above the zone of foliar application in Mn‐deficient YFEL. Black bars show leaves that received deionized water and Tween‐20 applications, while gray bars show leaves that received foliar Mn applications. Error bars show SD (*n* = 3).
**Figure S9** Leaf Mn concentrations for Mn‐sufficient (black bars) and Mn‐deficient (white bars) treated regions of YFELs following a 6 h foliar Mn application. Error bars indicate SD (*n* = 3). Control Mn‐sufficient leaves remained above the 15 μg g^−1^ DM threshold for bulk Mn sufficiency at both tillering (65 ± 10 μg g^−1^ DM) and flag leaf emergence (36 ± 8 μg g^−1^ DM), while control Mn‐deficient leaves were close to or below the threshold at tillering (6.7 ± 1 μg g^−1^ DM) and flag leaf emergence (16 ± 11 μg g^−1^ DM).
**Figure S10** Replicate *F*_v_/*F*_m_ PAM assays of physiological plant Mn status in YFEL at tillering and flag leaf emergence. Transects were measured through a linear zone of foliar applied droplets parallel to the leaf margin, from below to above the treated leaf region, in Mn‐deficient leaves with foliar Mn applications (black line), control Mn‐sufficient leaves (gray line), and control Mn‐deficient leaves (gray dashed line). Horizontal dotted lines indicate mild (0.75–0.6), moderate (0.6–0.5) or severe (<0.5) Mn‐deficiency.
**Figure S11** *F*_v_/*F*_m_ PAM assay of physiological plant Mn status in Mn‐sufficient YFEL after 6 h of foliar MnSO_4_ application. Colored scale bar shows *F*_v_/*F*_m_ values from 0 to 1. Dotted lines indicate zone of foliar application.
**Figure S12** Representative LA‐ICP‐MS scans of P‐deficient YFEL cross‐sections at flag leaf emergence, where (a, d) are the bright field microscopy images, (b, e) show the ^31^P elemental distributions and (c, f) show ^51^V elemental distributions. White scale bars indicate 200 μm for microscopy images. Elemental scale bars indicate counts (signal intensity) (NB: 0–0.6 counts (^51^V) for the control, 0–8 counts (^51^V) for the foliar PV treatment). BSE = bundle sheath extension, FC = fiber cell, M = mesophyll, T = trichome, VB = vascular bundle.
**Figure S13** Representative LA‐ICP‐MS scans of P‐deficient YFEL cross‐sections at flag leaf emergence, where (a, d) are the bright field microscopy images, (b, e) show the ^31^P elemental distributions and (c, f) show ^51^V elemental distributions. White scale bars indicate 200 μm for microscopy images. Elemental scale bars indicate counts (signal intensity) (NB: 0–0.45 counts (^51^V) for the control, 0–14 counts (^51^V) for the foliar PV treatment). BSE = bundle sheath extension, FC = fiber cell, M = mesophyll, T = trichome, VB = vascular bundle.
**Figure S14** Leaf V concentrations measured by ICP‐MS in leaf regions below, containing and above the zone of foliar application. Black bars indicate leaves that received foliar water applications, while gray bars show leaves that received foliar PV applications. Error bars show SD (*n* = 3).
**Figure S15** Leaf V concentrations for P‐sufficient (black) and P‐deficient (gray) treated regions of YFELs following a 6 h foliar PV application. Error bars indicate SD (*n* = 3). Control leaves had low background V concentrations at both growth stages (maximum concentration 0.46 ± 0.2 μg g^−1^ DM).
**Figure S16** Replicate NPQ PAM assays of physiological plant P status in YFEL at tillering and flag leaf emergence. Transects were measured through a zone of foliar applied droplets parallel to the leaf margin, from below to above the treated leaf region, in P‐deficient leaves with foliar PV applications (black line), control P‐sufficient leaves (gray line) and control P‐deficient leaves (gray dashed line). The horizontal dotted line indicates the approximate threshold for P‐deficiency (>0.8).
**Figure S17** NPQ PAM assay of physiological plant P status in P‐sufficient YFEL after 6 h of foliar PV application. Colored scale bar shows NPQ values from 0 to 4. Dotted lines indicate zone of foliar application.
**Table S1** Required concentration of foliar‐applied Mn vs P solutions to be absorbed to raise tissue concentrations to the “sufficient” nutrient thresholds (per 5 mm^2^ leaf area below droplet)

## Data Availability

Data can be provided upon request to the corresponding author.
