# Supplementary material for: Foliar‐applied manganese and phosphorus in deficient barley: Linking absorption pathways and leaf nutrient status
Source: Physiol Plant. 2022 Aug 22;174(4):e13761. doi: 10.1111/ppl.13761 (PMC9543583; doi:10.1111/ppl.13761)
Supplement: Supplementary file 1 — Figure S1 Leaf concentrations of (a) phosphorus and (b) manganese. Black bars show control leaves (P‐ and Mn‐sufficient), gray bars show P‐deficient leaves and white bars show Mn‐deficient leaves. Dotted lines indicate published thresholds for P‐deficiency (2000 μg g−1 DM) and Mn‐deficiency (15 μg g−1 DM). Error bars show SD (n = 3). Figure S2 Control (P‐sufficient and Mn‐sufficient), P‐deficient and Mn‐deficient barley plants at tillering (21 DAS) and following flag leaf emergence (51 DAS). Figure S3 YFEL leaf indices by nutrient deficiency and growth stage at tillering (T) or flag leaf emergence (FLE). (a) Specific leaf area and (b) leaf dry matter content for control (black bars), P‐deficient (gray bars) and Mn‐deficient (white bars) plants. Error bars show SD (n = 9). Figure S4 Total dry biomass production for control (black bars), P‐deficient (gray bars) and Mn‐deficient plants (white bars) at 51 DAS. (a) Shoot weight; (b) root weight; (c) root: shoot ratio. Error bars show SD (n = 6). Figure S5 ATR‐FTIR peak area ratio of wavenumbers representing (a) cellulose (3300–3350 cm−1) and (b) cellulose and noncellulosic structural polysaccharides (NCSPs) (1020–1056 cm−1), each relative to the peak area of holocellulose (895 cm−1) for control, P‐deficient and Mn‐deficient YFEL adaxial scans. Black bars represent YFEL at tillering, gray bars represent YFEL at flag leaf emergence. Data points show the mean and error bars show SD (n = 3). Different letters indicate statistically significant differences between means (ANOVA, α = 0.05). Figure S6 ATR‐FTIR peak area ratio of wavenumbers representing the (a) CH2 asymmetric band (2945–2866 cm−1) and (b) CH2 symmetric band (2866–2820 cm−1), each relative to the peak area of holocellulose (895 cm−1) for control, P‐deficient and Mn‐deficient YFEL adaxial scans. Black bars represent YFEL at tillering, gray bars represent YFEL at flag leaf emergence. Data points show the mean and error bars show SD (n = 3). Asterisks indicate stati [file PPL-174-0-s001.docx]

## Appendix A: Supplementary Information

Authors: Arsic M, Persson DP, Schjoerring JK, Thygesen LG, Lombi E, Doolette CL, Husted S

**
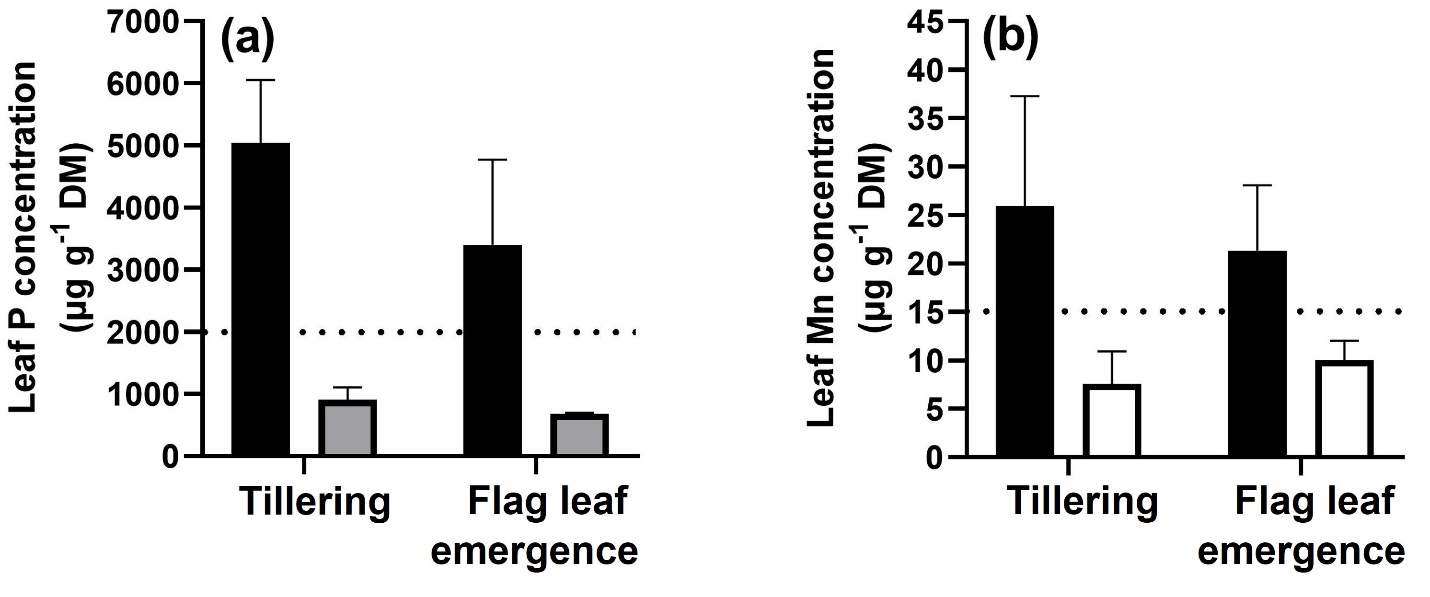
**

**Fig. S1** Leaf concentrations of (a) phosphorus and (b) manganese. Black bars show control leaves (P- and Mn-sufficient), grey bars show P-deficient leaves and white bars show Mn-deficient leaves. Dotted lines indicate published thresholds for P-deficiency (2000 µg g-1 DM) and Mn-deficiency (15 µg g-1 DM). Error bars show SD (n=3).

**
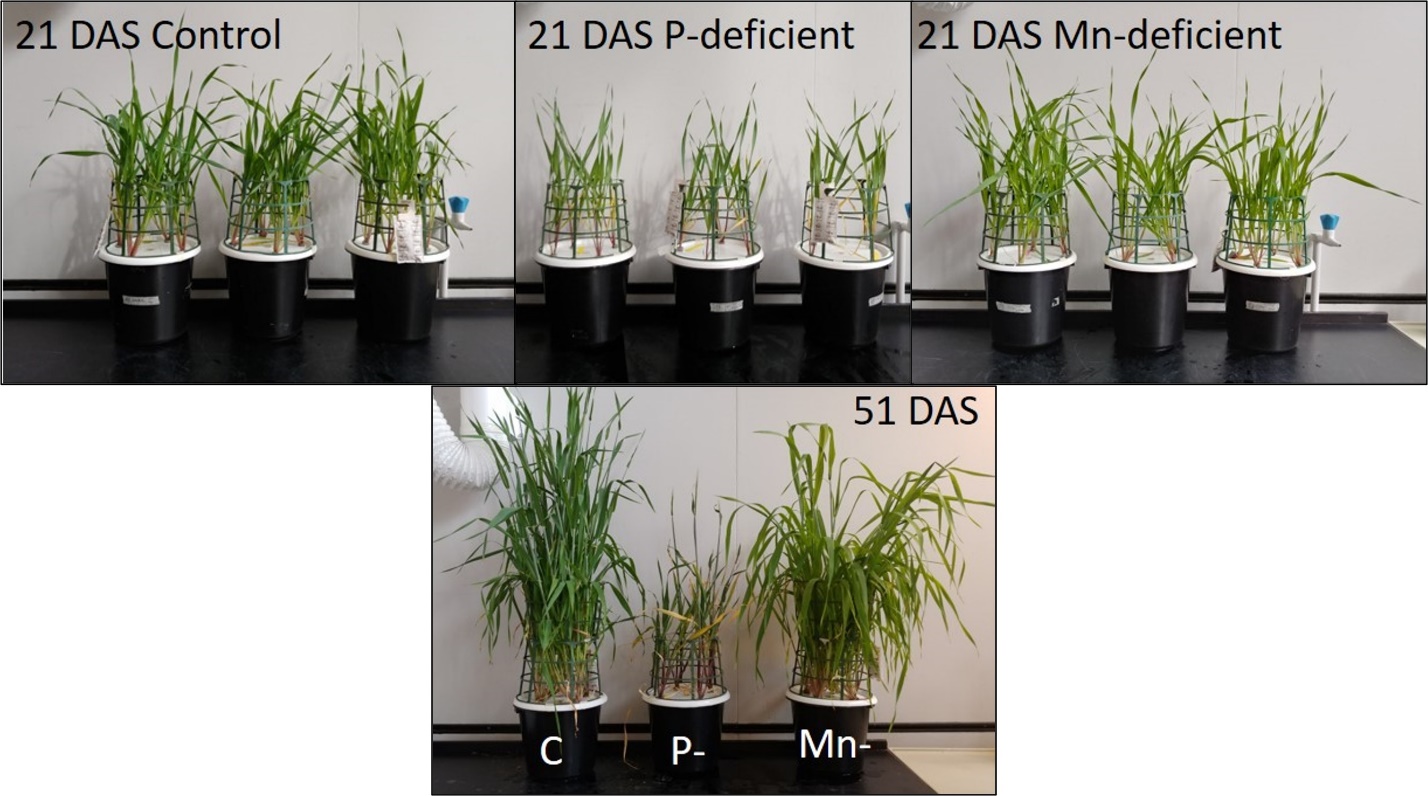
**

**Fig. S2** Control (P-sufficient and Mn-sufficient), P-deficient and Mn-deficient barley plants at tillering (21 DAS) and following flag leaf emergence (51 DAS).

**
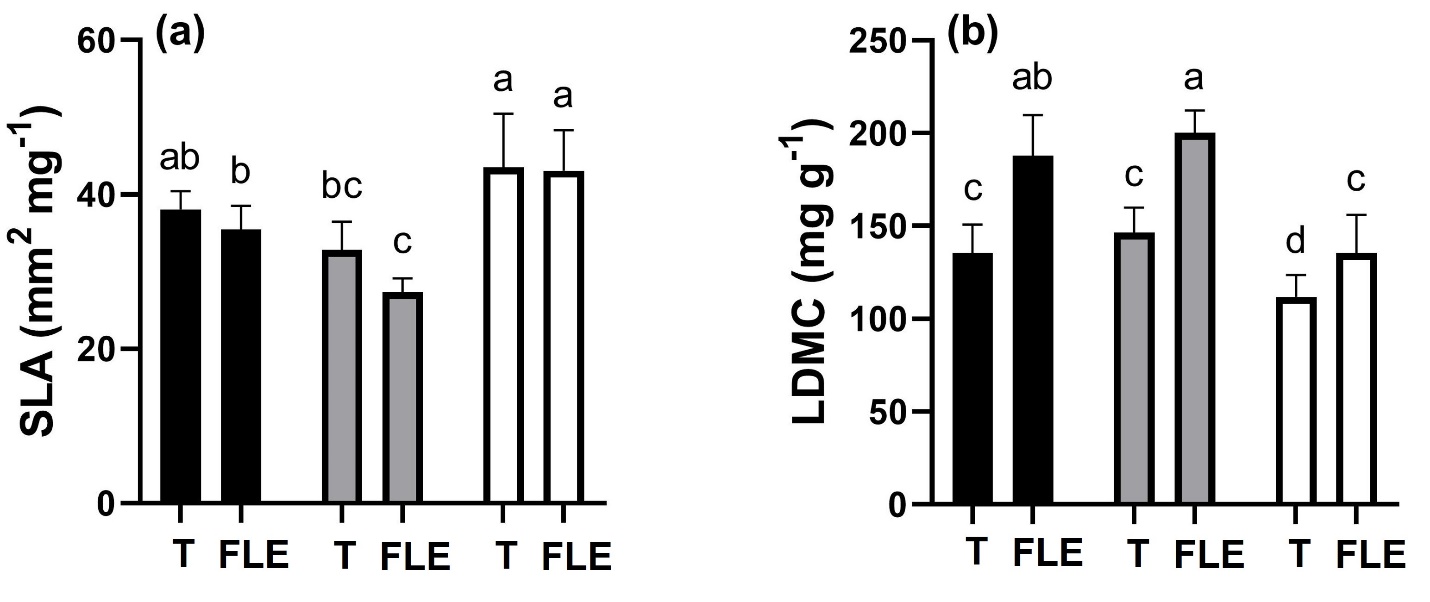
**

**Fig. S3** YFEL leaf indices by nutrient deficiency and growth stage at tillering (T) or flag leaf emergence (FLE). (a) Specific leaf area and (b) leaf dry matter content for control (black bars), P-deficient (grey bars) and Mn-deficient (white bars) plants. Error bars show SD (n=9).

**
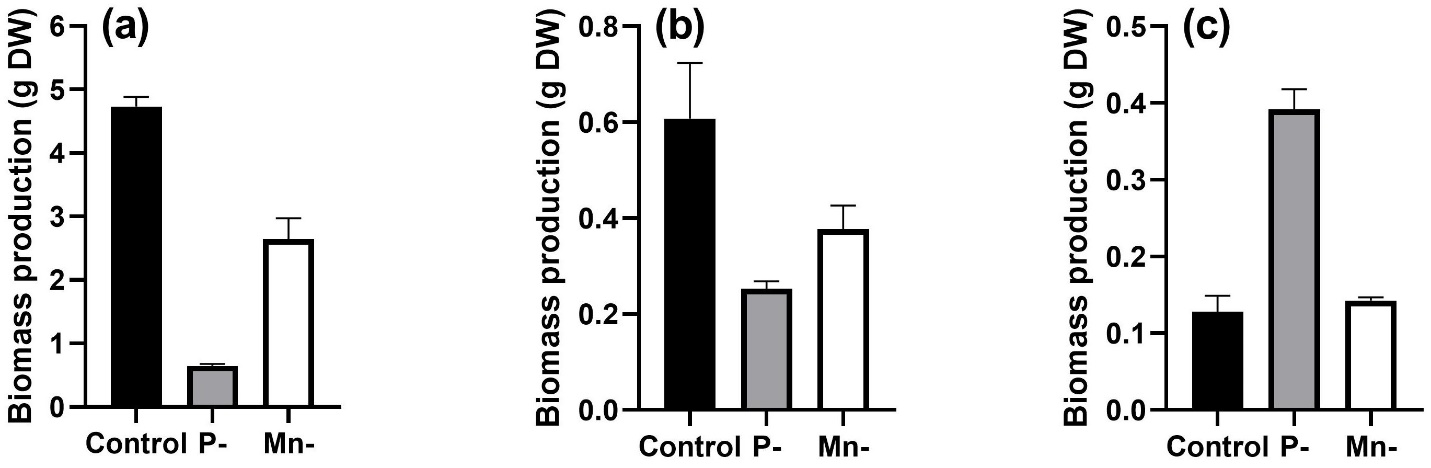
**

**Fig. S4** Total dry biomass production for control (black bars), P-deficient (grey bars) and Mn-deficient plants (white bars) at 51 DAS. (a) shoot weight; (b) root weight; (c) root: shoot ratio. Error bars show SD (n=6).

**
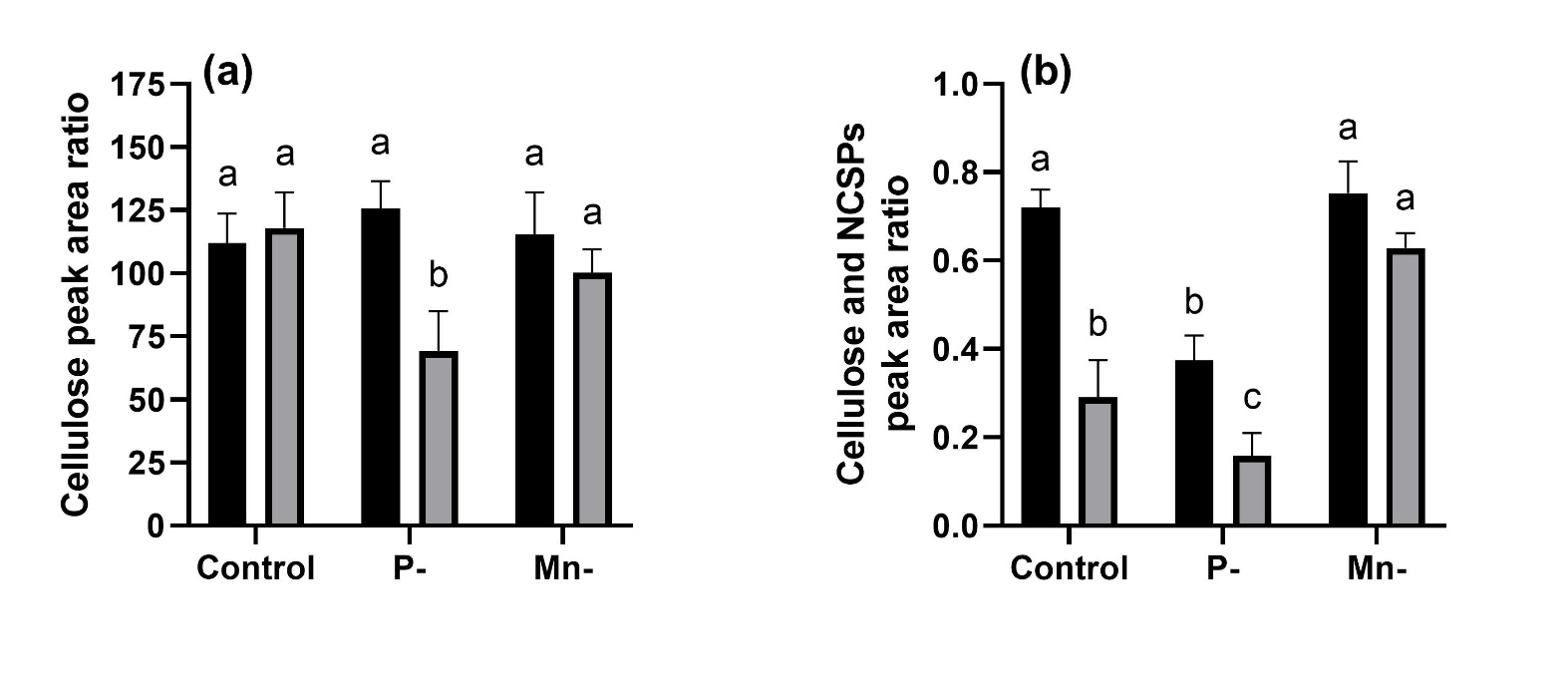
**

**Fig. S5** ATR-FTIR peak area ratio of wavenumbers representing (a) cellulose (3300 – 3350 cm^-1^) and (b) cellulose and non-cellulosic structural polysaccharides (NCSPs) (1020 – 1056 cm^-1^), each relative to the peak area of holocellulose (895 cm^-1^) for control, P-deficient and Mn-deficient YFEL adaxial scans. Black bars represent YFEL at tillering, grey bars represent YFEL at flag leaf emergence. Data points show the mean and error bars show SD (n=3). Different letters indicate statistically significant differences between means (ANOVA, α = 0.05).

**
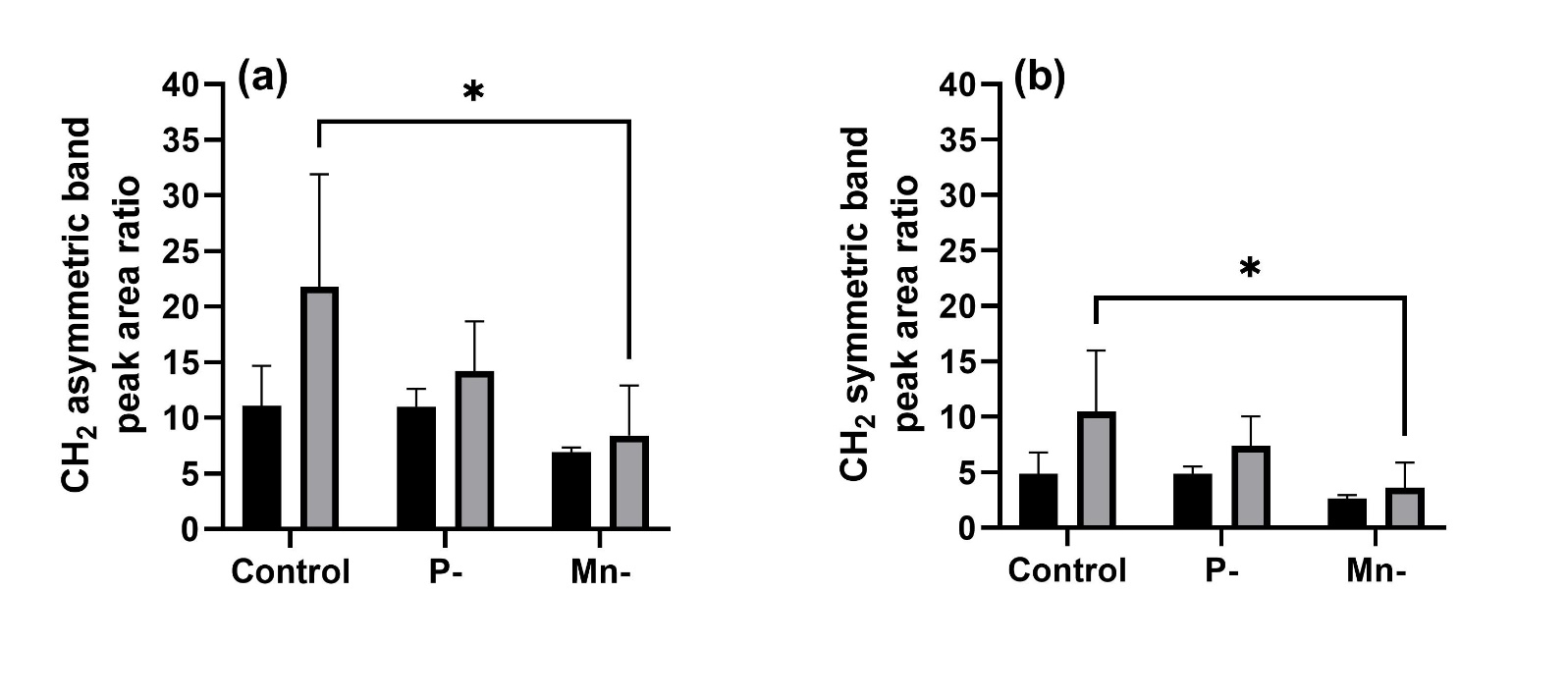
**

**Fig. S6** ATR-FTIR peak area ratio of wavenumbers representing the (a) CH_2_ asymmetric band (2945 – 2866 cm^-1^) and (b) CH_2_ symmetric band (2866 – 2820 cm^-1^), each relative to the peak area of holocellulose (895 cm^-1^) for control, P-deficient and Mn-deficient YFEL adaxial scans. Black bars represent YFEL at tillering, grey bars represent YFEL at flag leaf emergence. Data points show the mean and error bars show SD (n=3). Asterisks indicate statistically significant differences between means (ANOVA, α = 0.05).

**
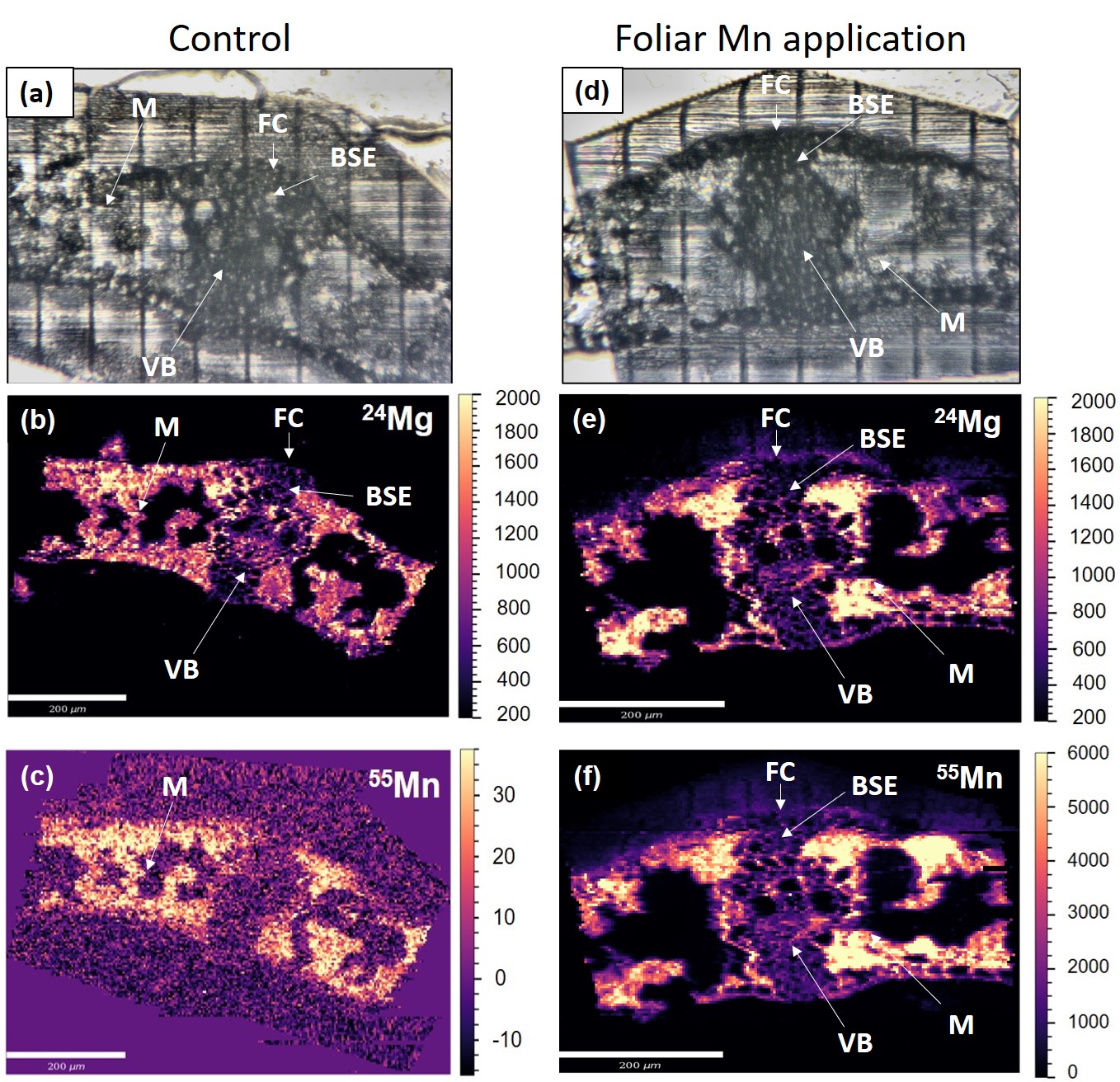
**

**Fig. S7** Representative LA-ICP-MS scans of Mn-deficient YFEL cross-sections at flag leaf emergence, where (a, d) are the bright field microscopy images, (b, e) show the ^24^Mg elemental distributions and (c, f) show ^55^Mn elemental distributions. White scale bars indicate 200 µm for microscopy images. Element scale bars indicate counts (signal intensity) (NB: 0-36 counts for the control, 0-2000 counts for the foliar Mn treatment). BSE = bundle sheath extension, FC = fiber cell, M = mesophyll, VB = vascular bundle.

**
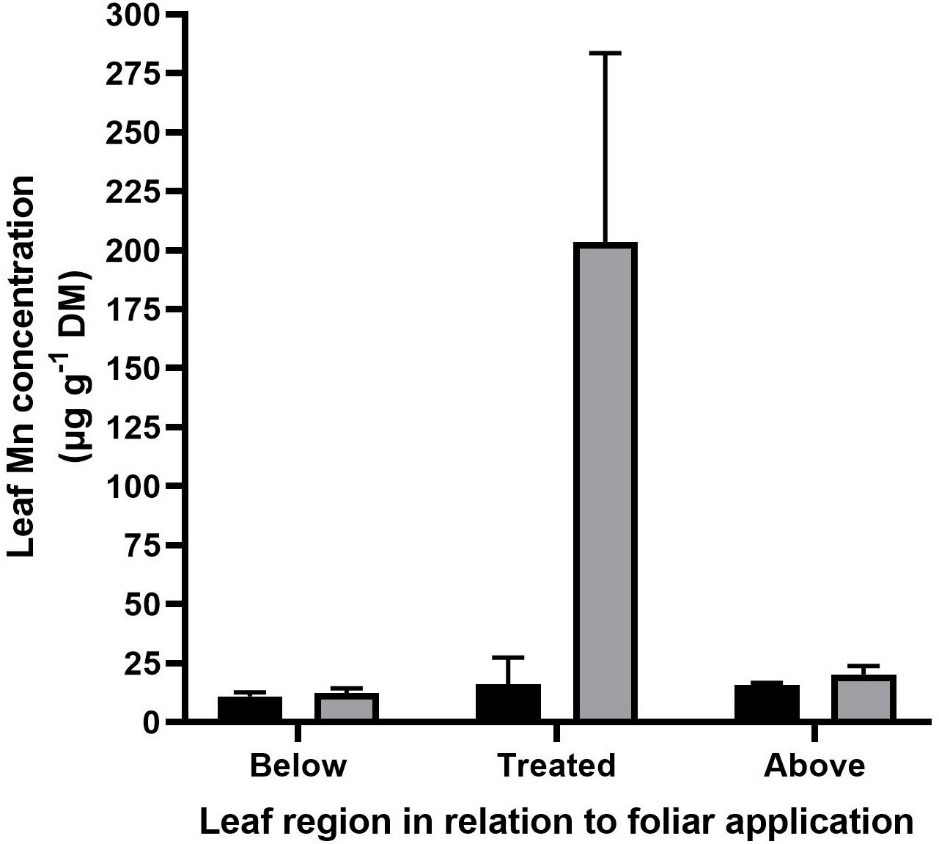
**

**Fig. S8** Leaf Mn concentrations measured by ICP-MS in leaf regions below, containing and above the zone of foliar application in Mn-deficient YFEL. Black bars show leaves that received deionised water and Tween-20 applications, while grey bars show leaves that received foliar Mn applications. Error bars show SD (n=3).

**
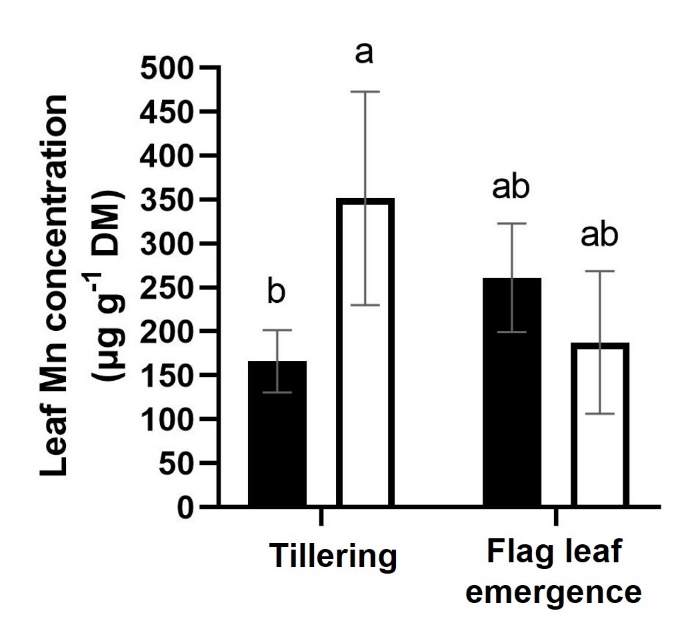
**

**Fig. S9** Leaf Mn concentrations for Mn-sufficient (black bars) and Mn-deficient (white bars) treated regions of YFELs following a 6 h foliar Mn application. Error bars indicate SD (n = 3). Control Mn-sufficient leaves remained above the 15 µg g-1 DM threshold for bulk Mn sufficiency at both tillering (65 ± 10 µg g-1 DM) and flag leaf emergence (36 ± 8 µg g-1 DM), while control Mn-deficient leaves were close to or below the threshold at tillering (6.7 ± 1 µg g-1 DM) and flag leaf emergence (16 ± 11 µg g-1 DM).

**
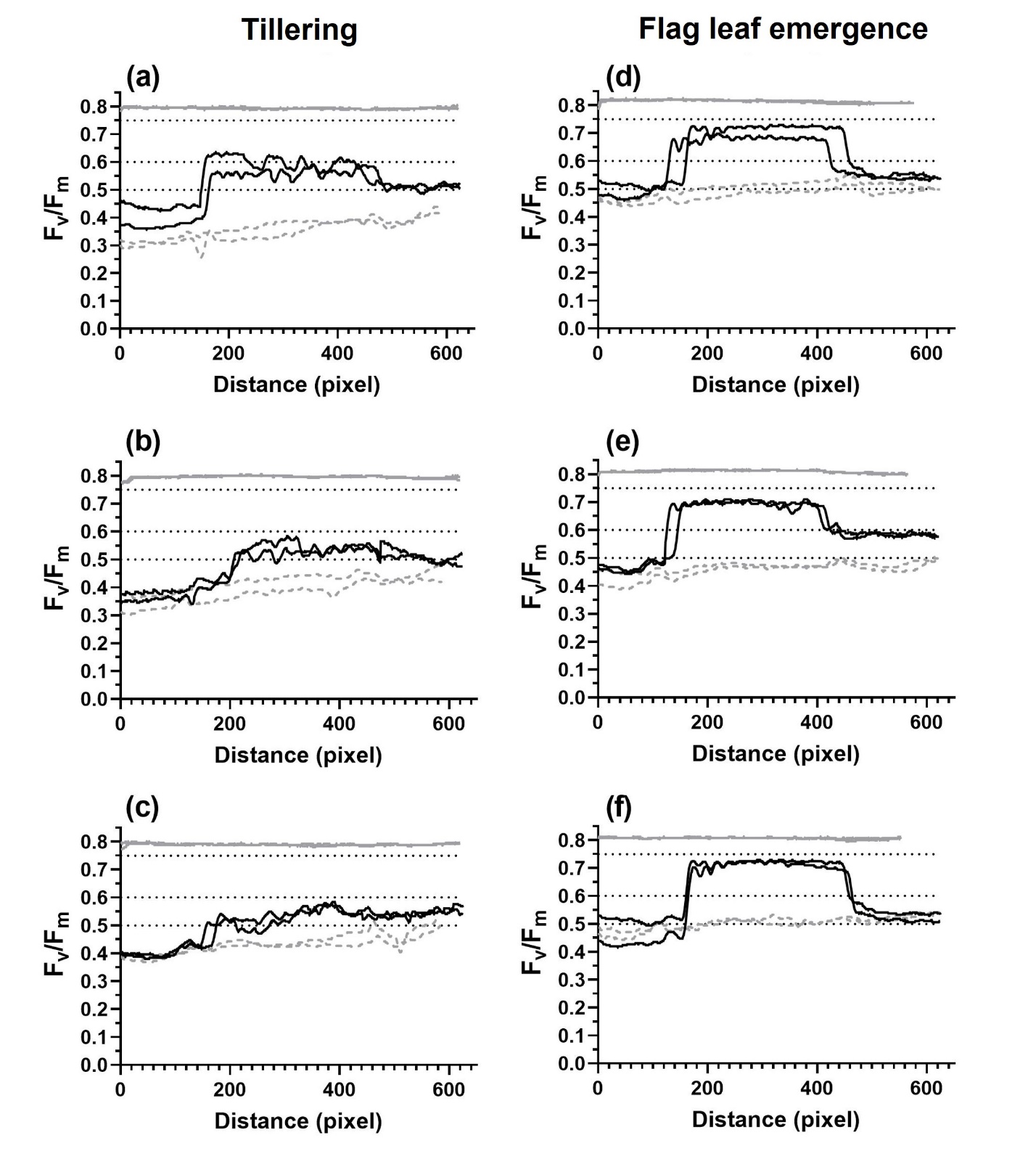
**

**Fig. S10** Replicate F_V_/F_M_ PAM assays of physiological plant Mn status in YFEL at tillering and flag leaf emergence. Transects were measured through a linear zone of foliar applied droplets parallel to the leaf margin, from below to above the treated leaf region, in Mn-deficient leaves with foliar Mn applications (black line), control Mn-sufficient leaves (grey line), and control Mn-deficient leaves (grey dashed line). Horizontal dotted lines indicate mild (0.75 – 0.6), moderate (0.6 – 0.5) or severe (<0.5) Mn-deficiency.

**
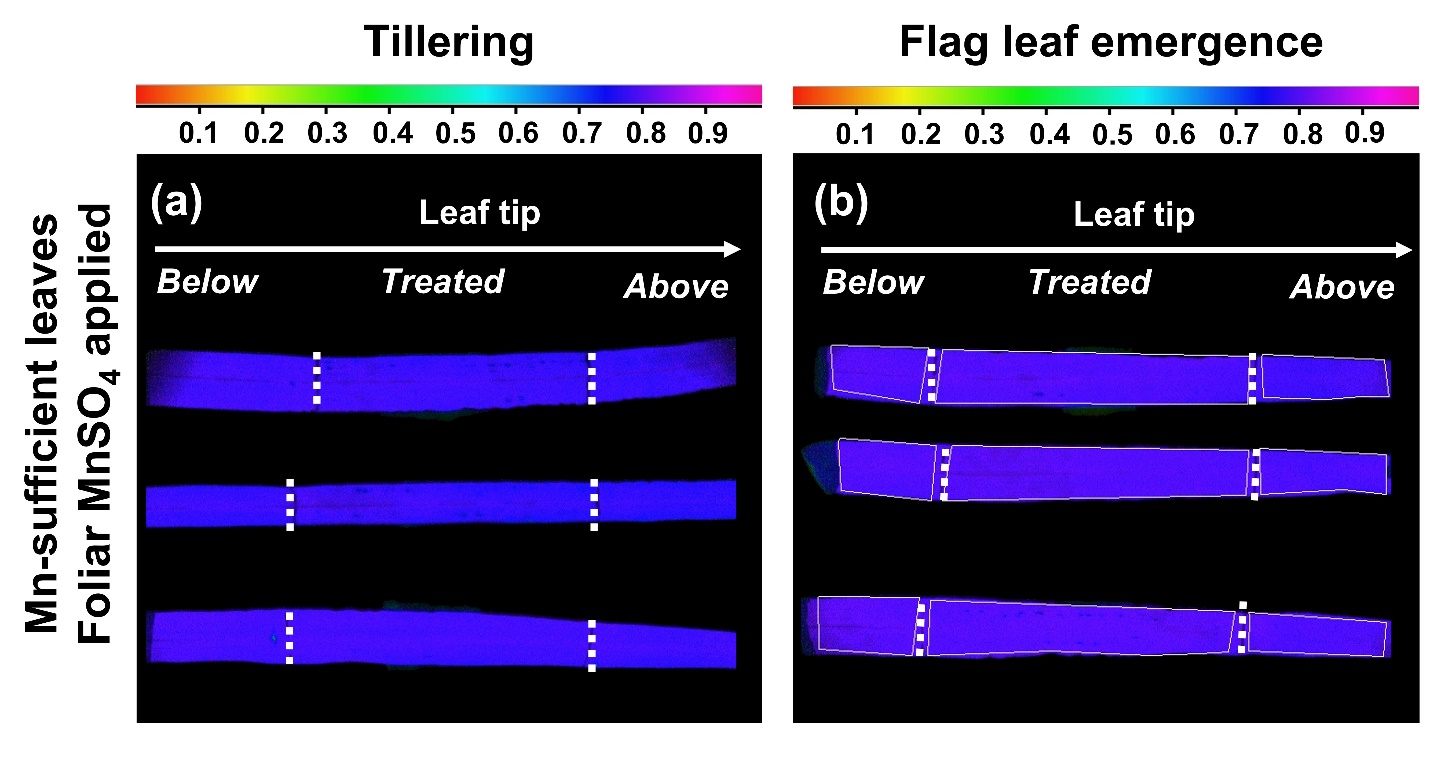
**

**Fig. S11** F_V_/F_M_ PAM assay of physiological plant Mn status in Mn-sufficient YFEL after 6 hours of foliar MnSO4 application. Coloured scale bar shows Fv/Fm values from 0-1. Dotted lines indicate zone of foliar application.

**
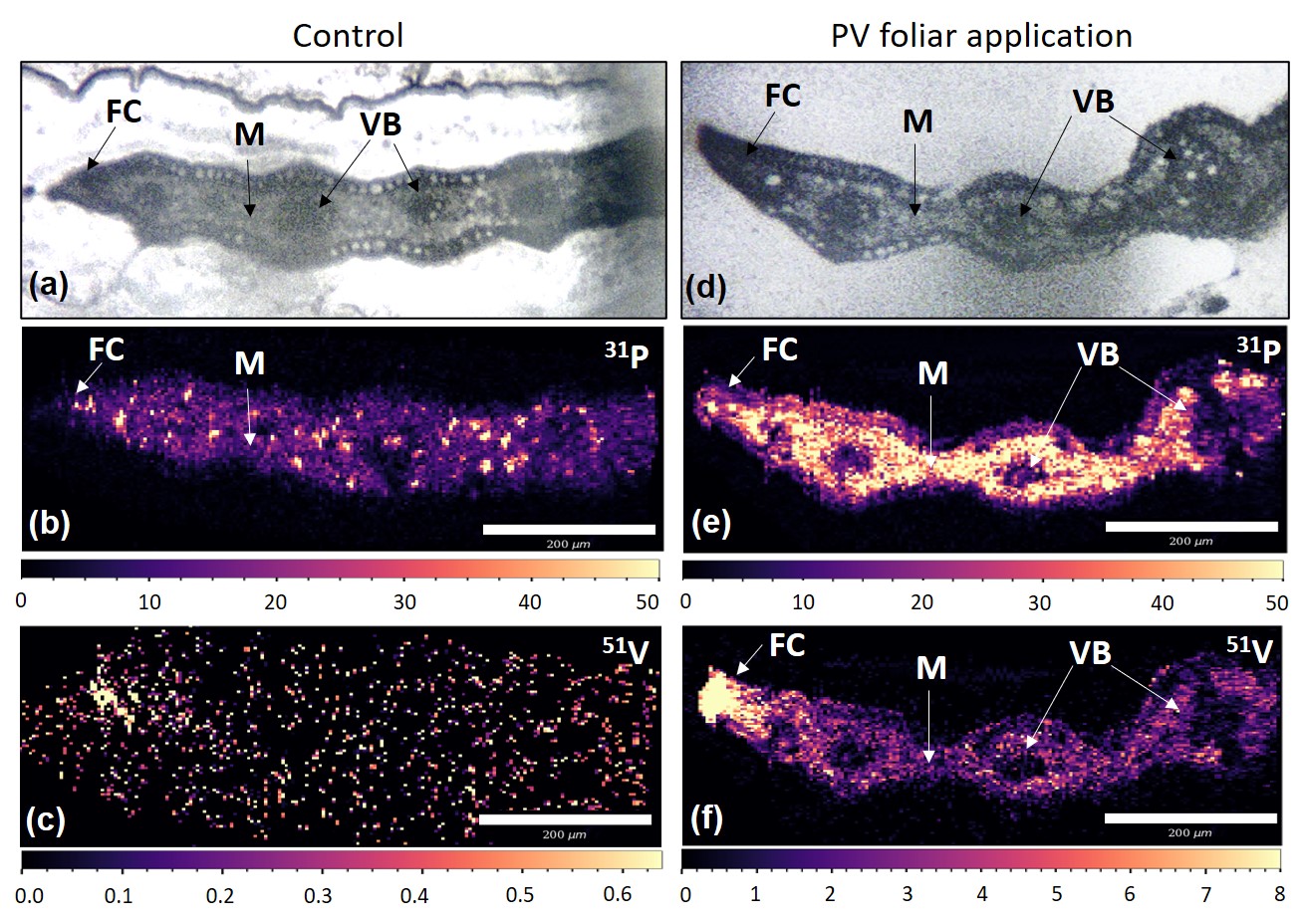
**

**Fig. S12** Representative LA-ICP-MS scans of P-deficient YFEL cross-sections at flag leaf emergence, where (a, d) are the bright field microscopy images, (b, e) show the ^31^P elemental distributions and (c, f) show ^51^V elemental distributions. White scale bars indicate 200 µm for microscopy images. Elemental scale bars indicate counts (signal intensity) (NB: 0-0.6 counts (^51^V) for the control, 0-8 counts (^51^V) for the foliar PV treatment). BSE = bundle sheath extension, FC = fiber cell, M = mesophyll, T = trichome, VB = vascular bundle.

**
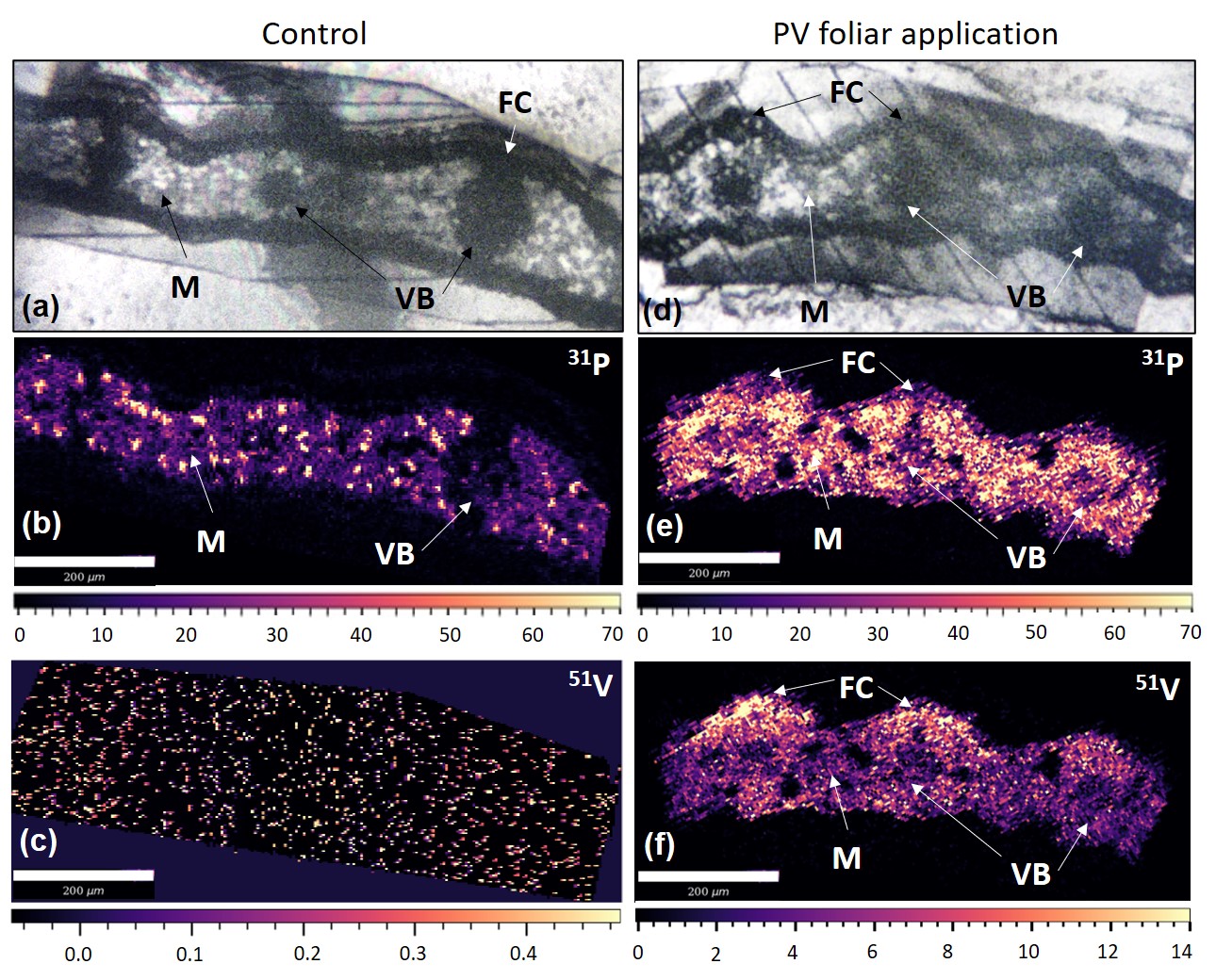
**

**Fig. S13** Representative LA-ICP-MS scans of P-deficient YFEL cross-sections at flag leaf emergence, where (a, d) are the bright field microscopy images, (b, e) show the ^31^P elemental distributions and (c, f) show ^51^V elemental distributions. White scale bars indicate 200 µm for microscopy images. Elemental scale bars indicate counts (signal intensity) (NB: 0-0.45 counts (^51^V) for the control, 0-14 counts (^51^V) for the foliar PV treatment). BSE = bundle sheath extension, FC = fiber cell, M = mesophyll, T = trichome, VB = vascular bundle.

**
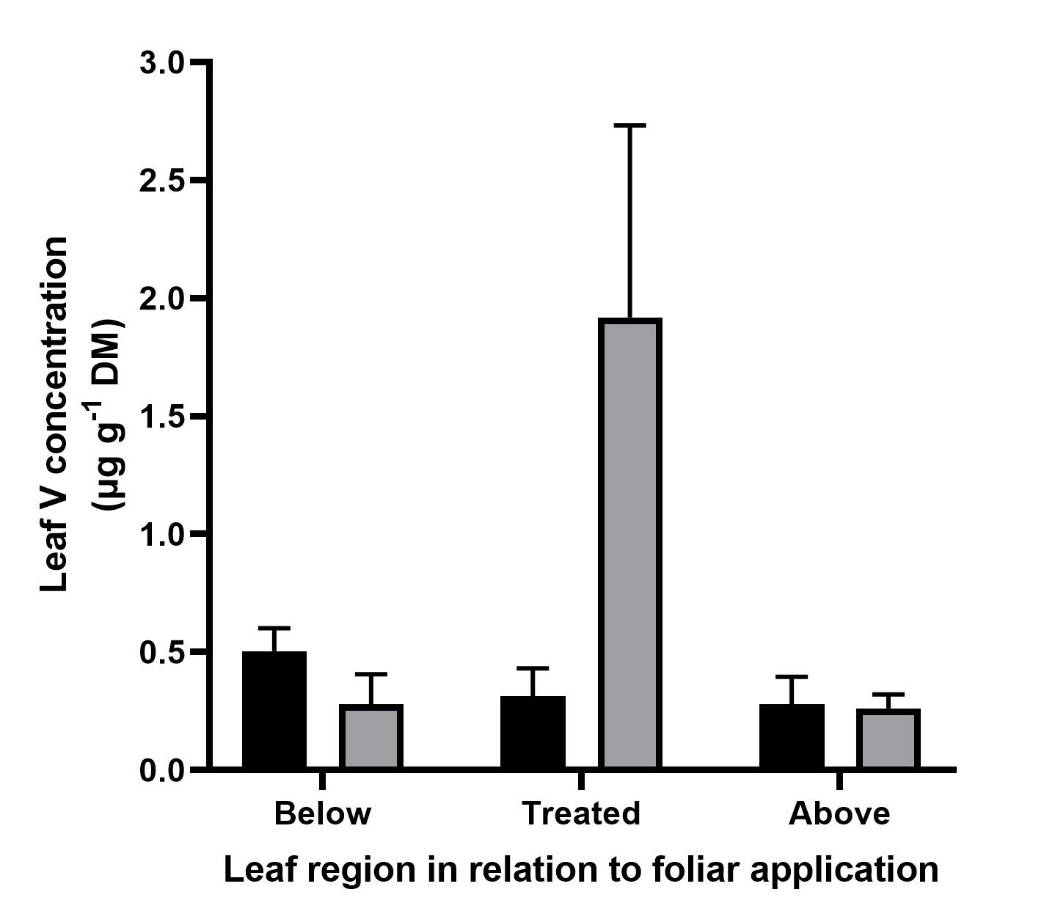
**

**Fig. S14** Leaf V concentrations measured by ICP-MS in leaf regions below, containing and above the zone of foliar application. Black bars indicate leaves that received foliar water applications, while grey bars show leaves that received foliar PV applications. Error bars show SD (n=3).

**
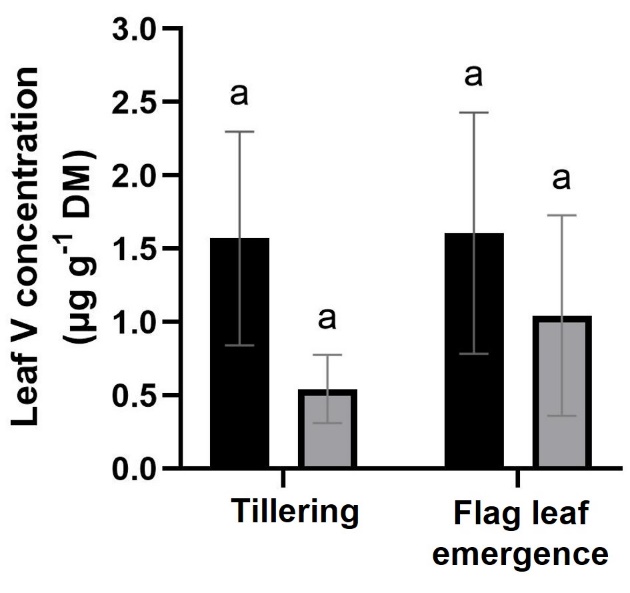
**

**Fig. S15** Leaf V concentrations for P-sufficient (black) and P-deficient (grey) treated regions of YFELs following a 6 h foliar PV application. Error bars indicate SD (n = 3). Control leaves had low background V concentrations at both growth stages (maximum concentration 0.46 ± 0.2 µg g^-1^ DM).

**
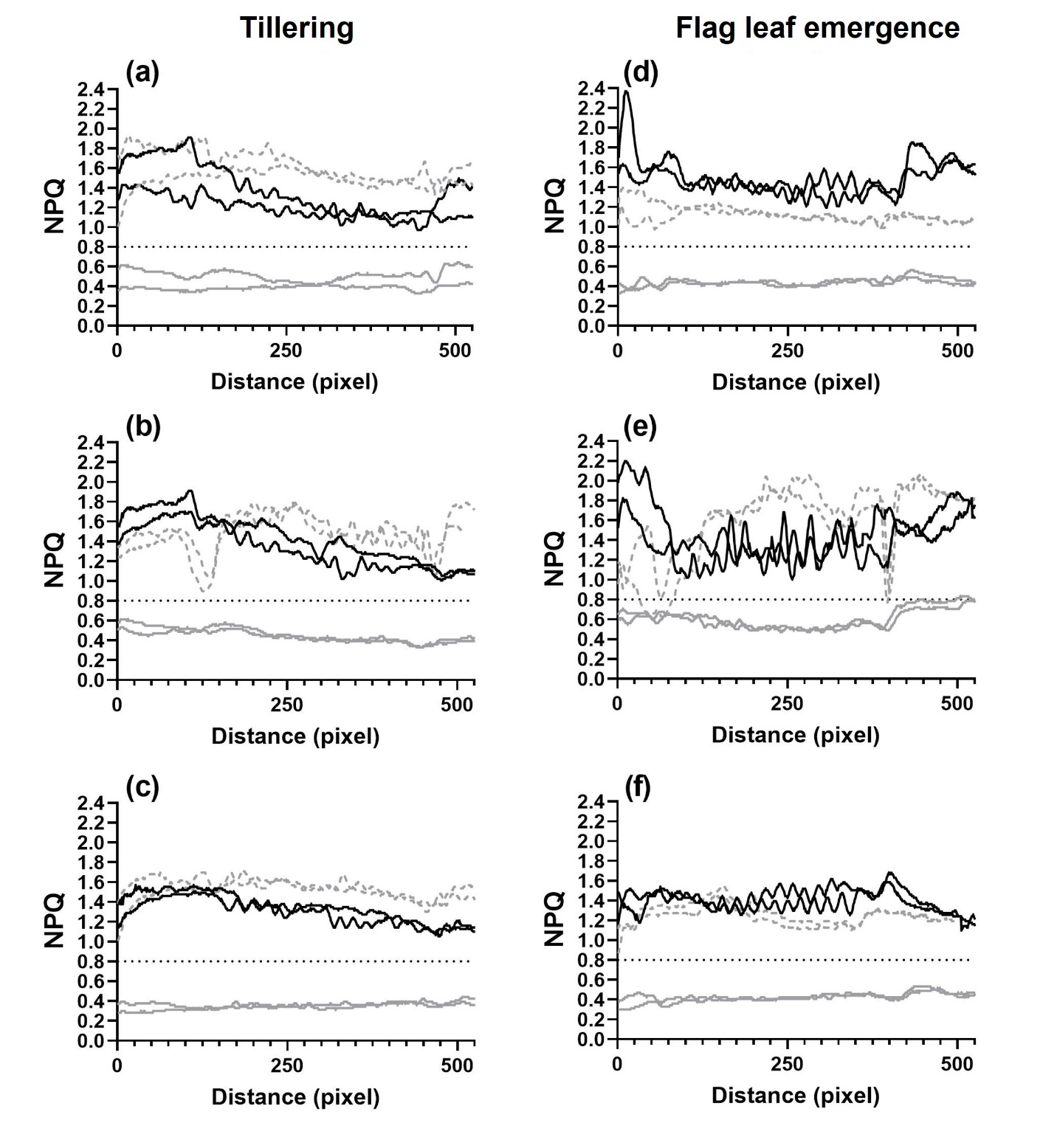
**

**Fig. S16** Replicate NPQ PAM assays of physiological plant P status in YFEL at tillering and flag leaf emergence. Transects were measured through a zone of foliar applied droplets parallel to the leaf margin, from below to above the treated leaf region, in P-deficient leaves with foliar PV applications (black line), control P-sufficient leaves (grey line) and control P-deficient leaves (grey dashed line). The horizontal dotted line indicates the approximate threshold for P-deficiency (> 0.8).

**
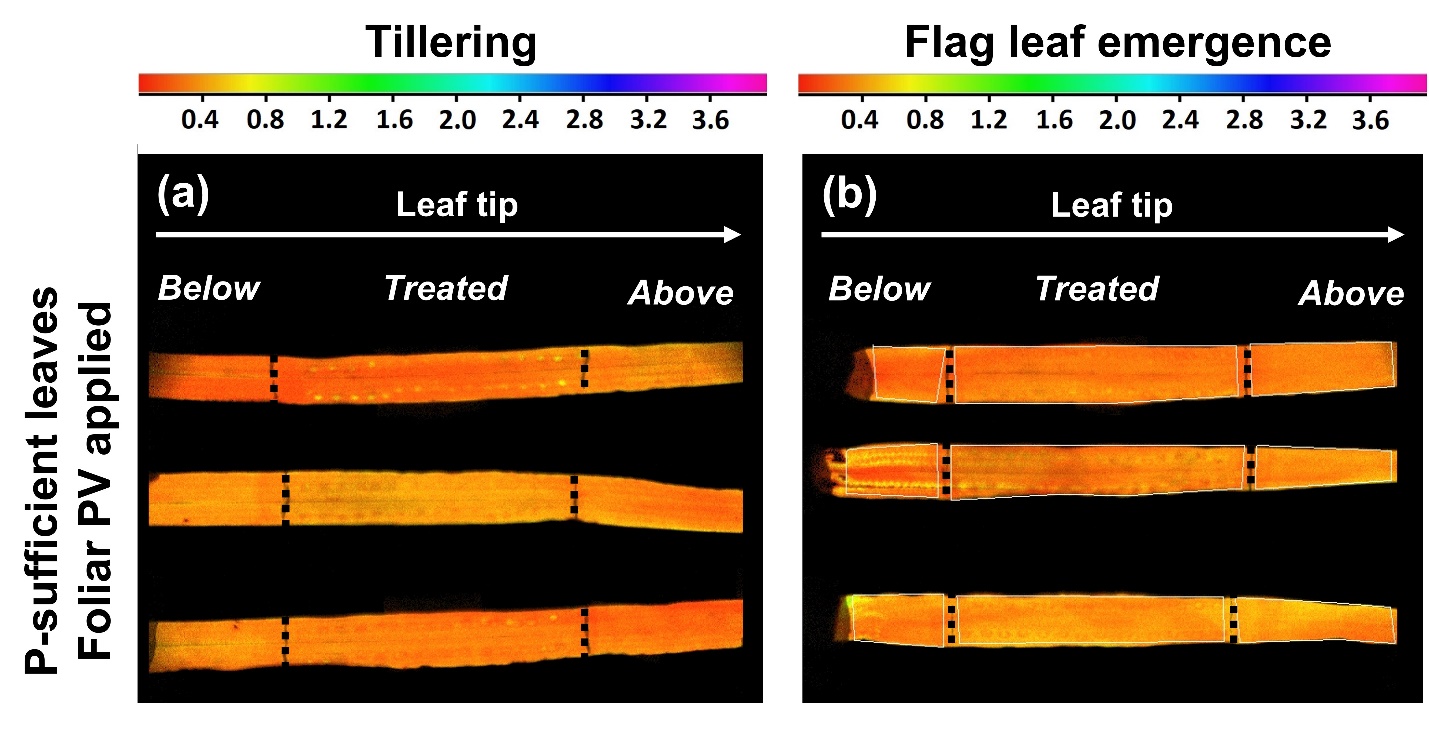
**

**Fig. S17** NPQ PAM assay of physiological plant P status in P-sufficient YFEL after 6 hours of foliar PV application. Coloured scale bar shows NPQ values from 0-4. Dotted lines indicate zone of foliar application.

**Table S1** Required concentration of foliar-applied Mn vs P solutions to be absorbed to raise tissue concentrations to the “sufficient” nutrient thresholds (per 5 mm^2^ leaf area below droplet)

| **Nutrient** | **Nutrient concentration in deficient YFEL**  **(µg g-1 DW)** | **Nutrient concentration in sufficient YFEL**  **(µg g-1 DW)** | **Mass nutrient applied per 5µL droplet (µg)** | **Mass leaf area covered by 5µL droplet (µg DW)** | **Amount of foliar-applied solution absorbed to reach nutrient sufficiency (%)** |
| --- | --- | --- | --- | --- | --- |
| **Mn** | 10 | 15 | 5.5 | 140 | 0.01 |
| **P** | 800 | 2000 | 31 | 200 | 0.8 |
